# *In Silico* Design and Immunogenicity Studies of Two Multi-Epitope Vaccines against *Klebsiella pneumoniae*

**DOI:** 10.34133/csbj.0232

**Published:** 2026-10-28

**Authors:** Marta Vicente-Pazos, Mireia López-Siles, Michael J. McConnell

**Affiliations:** ^1^ Intrahospital Infections Laboratory, National Centre for Microbiology, Instituto de Salud Carlos III (ISCIII), Madrid, Spain.; ^2^ Doctoral Program in Biomedical Sciences and Public Health, IMIENS, Universidad Nacional de Educación a Distancia (UNED), Madrid, Spain.; ^3^ Microbiology of Intestinal Diseases, Biology Department, Universitat de Girona, Girona, Spain.; ^4^ Department of Biological Sciences, University of Notre Dame, Notre Dame, IN, USA.; ^5^ Eck Institute for Global Health, Notre Dame, IN, USA.

## Abstract

*Klebsiella pneumoniae* represents an important clinical problem worldwide, causing infections in healthy and immunocompromised individuals that are often resistant to multiple antibiotics. The diversity of potential antigens encoded by *K. pneumoniae* makes the development of a protein-based vaccine that combines different antigens a promising strategy to achieve high vaccine coverage. This study employs 8 highly conserved virulence factors encoded by *K. pneumoniae* to construct multi-epitope vaccines. The target proteins used here are the fimbrial subunit MrkA, the outer membrane proteins and porins OmpA, OmpW, LamB, and PhoE, and the siderophore receptors FepA, FhuA, and Fiu. Two different immunoinformatics strategies (BEPI and VAXI) were followed to select one B cell epitope per protein for vaccine construct design. Whereas the BEPI-strategy prioritizes the B cell epitope prediction score, the VAXI-strategy gives importance to B cell epitope antigenicity score. The validated multi-epitope sequences were fused to the immunostimulatory PADRE peptide. BEPI- and VAXI-vaccine proteins were predicted to be stable and easily expressed in *Escherichia coli* and *Homo sapiens*. Moreover, both met the safety thresholds of non-allergenicity, non-toxicity, and non-human homology. Specifically, the VAXI-vaccine protein scored higher for solubility and antigenicity and was predicted to induce improved humoral and cellular responses. The predicted secondary structures of both vaccines consisted of >70% random coils, >15% α-helices, and ~10% β-strands, and their predicted 3-dimensional models indicated that most regions were exposed. Finally, >99.9% of global population coverage was predicted for both multi-epitope vaccines. We thus present 2 multi-epitope vaccines against *K. pneumoniae* virulence factors with promising physicochemical profiles regarding production and potential safety for preclinical testing and eventual use in humans.

## Highlights

• 44 conserved B cell epitopes from 8 *K. pneumoniae* virulence factors were identified.
• Two multi-epitope vaccine constructs were predicted to be highly antigenic.
• Multi-epitope vaccine constructs were sequence optimized and predicted to be stable.
• Constructs were predicted to be non-homologous, non-allergenic, and non-toxic human peptides.
• Both multi-epitope vaccines were predicted to elicit humoral and cellular responses.

## Graphical Abstract

**Figure:**
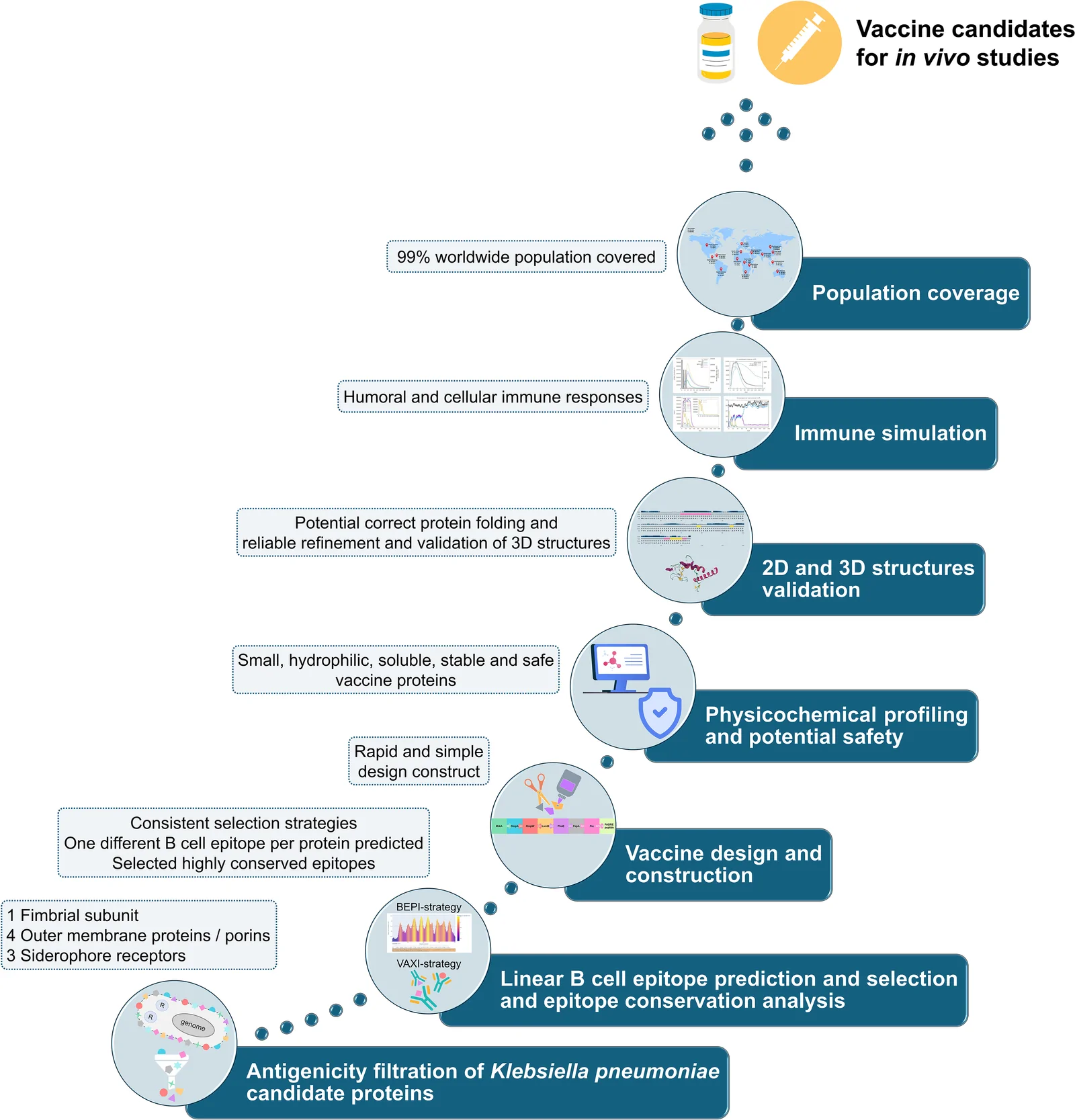

## Introduction

*Klebsiella pneumoniae* is a non-motile, encapsulated Gram-negative bacillus, and a commensal microorganism in different parts of the human body, but can also cause infection in both immunocompromised and immunocompetent individuals. It is one of the most commonly isolated nosocomial pathogens [1,2], often as the causative agent of pneumonia, urinary tract infections, and bacteremia [1,3,4]. *K. pneumoniae* can acquire and transmit plasmids harboring antimicrobial resistance genes or virulence factors, acquiring a multidrug-resistant [5] or hypervirulent phenotype [6], or even developing a combined phenotype [7–9]. This profile of *K. pneumoniae* leads to the development of more severe infections (such as endophthalmitis, meningitis, liver abscess, or soft tissue infections) and severely complicates its treatment with antibiotics (broad- and extended-spectrum β-lactams, including carbapenems [3,10], and colistin [10,11]). This situation is particularly concerning for pediatric populations in low- and middle-income countries, where the morbidity and mortality rates associated with neonatal sepsis are increasing due to the high prevalence of third-generation cephalosporin resistance [12].

In 2019, a global burden analysis of infections due to antibiotic resistance revealed that more than 650,000 deaths per year worldwide are associated with *K. pneumoniae* infections, indicating that this pathogen is the second leading cause of global deaths attributed to infections caused by antibiotic-resistant species [13,14]. In this scenario, the latest report from the World Health Organization (WHO) includes *K. pneumoniae* within the category of critical priority pathogens as one of the most worrisome antibiotic-resistant species, requiring the exploration of alternative strategies, such as vaccination [15]. Active or passive immunization has the potential to be a cost-effective and accessible alternative to conventional antimicrobial therapy as development of vaccines against this pathogen can help prevent infections and reduce the associated mortality and morbidity [16].

The number of studies aiming to develop vaccines against *K. pneumoniae* has increased substantially during the last decade, and several attempts using different technological approaches have been reported [17–19]. However, only a few vaccines have reached clinical trials to date [13,17,19,20]. Reasons for the limited success of *K. pneumoniae* vaccines include the potential toxicity and allergenicity of some components, such as capsular polysaccharides (CPSs), lipopolysaccharides (LPSs), extracellular vesicles or certain antigenic proteins, and their low immunogenicity, which leads to the need for complex bioconjugate formulations [13,17–19,21]. Furthermore, the high variability in *K. pneumoniae* capsular serotypes potentially limits vaccine protection, as demonstrated by different CPS- and LPS-based vaccines tested in human trials [22–25]. A recent vaccine against *K. pneumoniae*, based on LPS-O antigens from the most predominant serotypes, entered phase I/II clinical trials in 2021, but limited data are available regarding the outcomes of this vaccine [18,20,26,27]. Another significant challenge in the translational progress of *K. pneumoniae* vaccine candidates is the lack of adequate animal models for preclinical safety, immunogenicity, and efficacy testing that accurately predict human responses [28]. The absence of a U.S. Food and Drug Administration (FDA)-approved vaccine that effectively prevents *K. pneumoniae* infections leads to the urgent requirement of new vaccine candidates for immune protection studies [29].

The development of immunoinformatics approaches alongside the constant improvement in different computational tools for *in silico* assessment and the availability of sequenced bacterial genomes allows the identification and study of novel potential antigens, as demonstrated by previous immunoinformatics studies of *K. pneumoniae* [30,31]. Different epitope-based vaccine strategies have been widely used for different microorganisms and diseases, including infectious bacteria (*Acinetobacter baumannii* [32–34], *Escherichia coli* [35,36], *Staphylococcus aureus* [37,38], *Chlamydia trachomatis* [39], or *Porphyromonas gingivalis* [40]), viruses [severe acute respiratory syndrome coronavirus 2 (SARS-CoV-2) [41–43], HIV [44], Marburgvirus [45], and Enterovirus [46]], fungi (*Candida auris* [47]), parasites (*Echinococcus granulosus* [48]), and different types of cancer including breast cancer [49,50], glioblastoma multiforme [51], or human papillomavirus and Wilms tumor [52].

Regarding *K. pneumoniae*, there are also several studies to date that use predicted B and T cell epitopes to construct multi-epitope vaccines [21,26,29,53]. In addition, there are other *in silico* vaccine approaches aimed at simultaneously targeting *K. pneumoniae* and other pathogens that cause co-infections, for example, *K. pneumoniae* and *Mycobacterium tuberculosis* [54], or *K. pneumoniae* and *Pseudomonas aeruginosa* [55]. In this regard, as multi-epitope vaccines combine epitopes from different antigens, the use of linear B cell epitopes may reduce the chance of misfolded sequences, resulting in less antigen degradation, and potentially increasing the ability of the antigen to induce a robust immune response. Importantly, antibody-mediated immunity remains the most extensively validated correlate of protection for extracellular *K. pneumoniae*, particularly through opsonizing antibodies directed against surface-exposed antigens.

Due to the great diversity among serotypes, pathogenicity, virulence, clinical incidence, and prevalence found in *K. pneumoniae* [2,3,13], vaccination strategies that combine different antigens from the target microorganism may represent a promising approach for achieving high vaccine coverage and preventing infections, in addition to reducing the carriage and spread of antimicrobial resistance [20]. Taking this into account, we aimed to construct a multi-epitope vaccine that incorporates epitopes from different *K. pneumoniae* virulence factors. The selected candidates included MrkA, the major scaffold subunit of type 3 fimbriae [56] that plays a role in adherence to abiotic surfaces [57], attachment to host cells [58], and biofilm formation [59,60]. It also included OmpA as the major and well-studied outer membrane protein already used in different types of vaccines [33,61–64], OmpW as a highly immunogenic protein in *Vibrio cholerae* [65,66] and in *A. baumannii* [67], and LamB and PhoE as porins that are overexpressed in carbapenem and β-lactam-resistant strains [68–72]. Lastly, FepA, FhuA, and Fiu were selected as siderophore receptors that capture different iron molecules, a very crucial factor during infection and colonization stages [73–77]. Collectively, these antigens are broadly conserved among *K. pneumoniae* lineages, including hypervirulent and multidrug-resistant strains, although their expression may vary with bacterial genotype, environmental conditions, and stage of infection. Importantly, several of the selected proteins, particularly the iron-acquisition receptors and the fimbrial component, are known to be induced during infection, supporting their biological relevance and expression during pathogenesis.

Recent studies, which have also used different proteins from *K. pneumoniae* or *P. aeruginosa* for multi-epitope vaccine design, have compared the position of the adjuvant [29], the use of different adjuvants [78–80], or antigen-specific and combined constructs [81]. In our study, we addressed 2 different strategies in epitope selection, considering the highest-scoring predicted epitopes as the most likely epitopes expected in physiological conditions, and the most antigenic epitopes as a common criterion followed in similar works. Our study distinguishes itself from previous *K. pneumoniae* multi-epitope vaccine designs in several key aspects. First, we present a direct comparative analysis of 2 epitope selection strategies (BEPI *vs*. VAXI), which has not been systematically evaluated in prior *K. pneumoniae* vaccine studies. While Hakimian *et al.* [29] compared adjuvant positions and Zhu *et al.* [80] examined different adjuvants in *P. aeruginosa*, our work specifically addresses whether prioritizing predicted B cell epitope scores *vs*. antigenicity scores yields vaccine candidates with improved predicted performance. Unlike previous work that use the same epitopes across vaccine constructs, we designed 2 multi-epitope vaccines based on distinct epitope sets. Because epitope composition is key for vaccine’s performance, this approach allowed us to investigate the potential of each epitope combination. Second, our protein selection strategically combines empirically validated protective antigens (MrkA, OmpA, and FepA) with computationally identified targets (LamB, PhoE, OmpW, and Fiu), providing both experimental grounding and expanded coverage. Third, we employ dual validation throughout our workflow, using at least 2 bioinformatics tools at each analytical step to enhance prediction reliability. This integrated comparative approach, coupled with our specific combination of virulence factors targeting 3 functional categories (fimbriae, porins, and iron acquisition systems), provides a distinctive contribution to *K. pneumoniae* vaccine immunoinformatics.

Most immunoinformatics studies are conducted with similar workflows. However, incomplete validation of the predicted epitopes [21,26,33,55,80,82] and the lack of population coverage analysis [21,26,82,83] or immune response simulation [29,83] are some of the main limitations of previous multi-epitope vaccines. In this regard and given that the novelty of this study lies in the epitope selection strategy, we validated the selected epitopes not only through classical sequence-based methods but also by considering their conformation as a 3-dimensional (3D) structure. Furthermore, our study compared the multi-epitope vaccines from their sequence-based physicochemical characteristics to solubility or aggregation propensity based on structural features and surface accessibility to approximate predictions to laboratory production conditions and their potential use *in vivo* [84]. In this sense, another approach of our work, absent in prior immunoinformatics efforts, is the optimization of the multi-epitope vaccine sequences to be expressed in *Homo sapiens* with a view to their use for other vaccine platforms (mRNA or DNA).

## Materials and Methods

### Selection of *K. pneumoniae* target proteins

The overall workflow followed for our multi-epitope vaccine design is presented in Fig. 1.

**Fig. 1.**
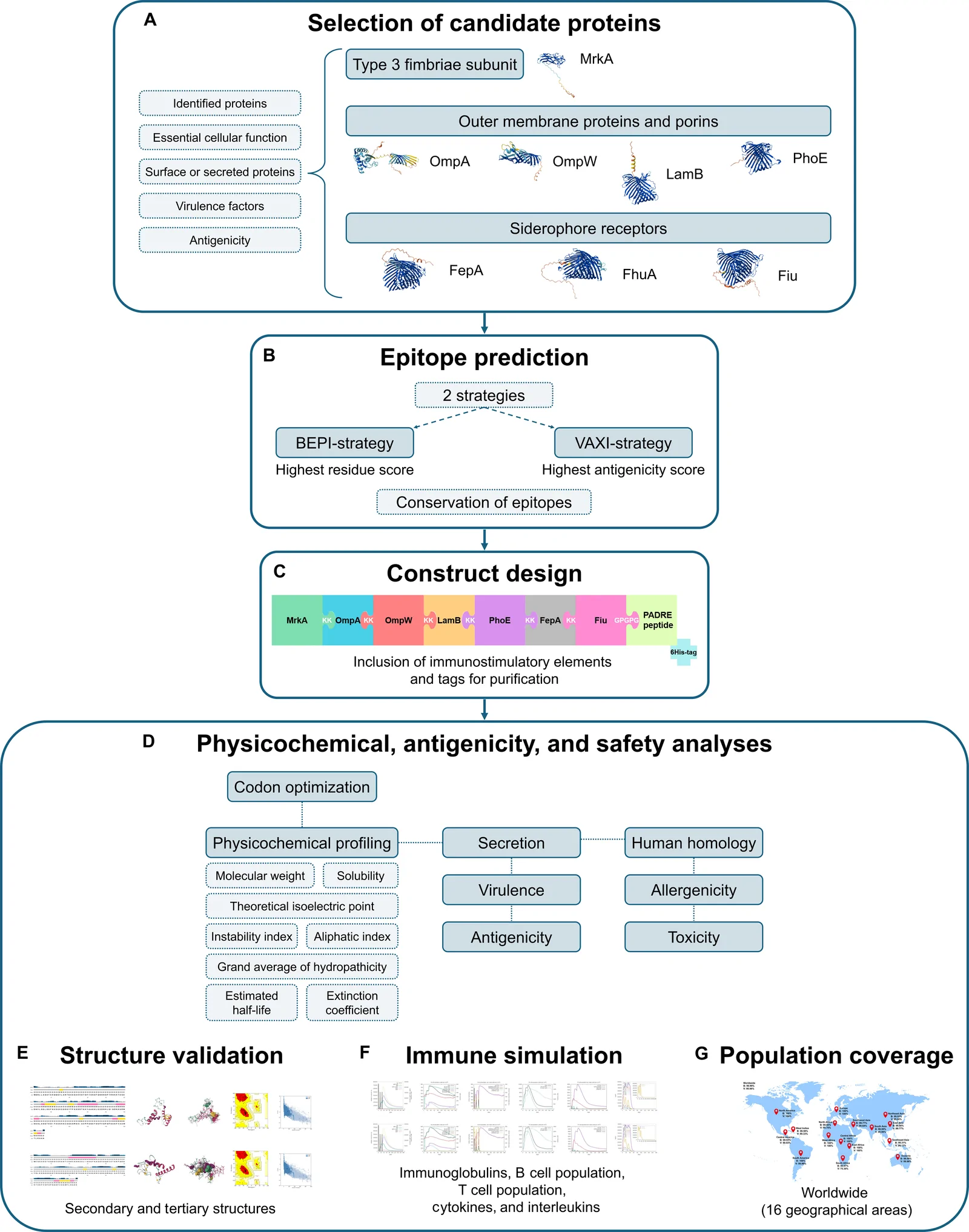
Study workflow. (A) Antigenic proteins of *Klebsiella pneumoniae* were selected at the first step for epitope prediction. (B) The second step comprised linear B cell epitope selection and validation with bioinformatics approaches. (C) The third step consisted in the multi-epitope vaccine construction. Lastly, (D) a physicochemical, antigenicity, and safety analysis and (E) a structure evaluation and validation were followed by (F) an immune simulation and (G) a population coverage analysis to complete the *in silico* study of the multi-epitope vaccines.

A total of 8 candidate proteins belonging to 3 different functional categories were selected from *K. pneumoniae* ATCC 43816, a hypervirulent and hyperencapsulated strain [85], or *K. pneumoniae* HS11286, a multidrug-resistant strain [86], to construct a multi-epitope vaccine. Specifically, we included the fimbriae protein MrkA (QPW33900.1), the outer membrane proteins and porins OmpA (QPW36129.1), OmpW (QPW35866.1), LamB (QPW32826.1), and PhoE (QPW32145.1), and the iron uptake proteins (also known as siderophore receptors) FepA (AEW58984.1), FhuA (QPW32251.1), and Fiu (QPW35864.1). The criteria for the selection of proteins included as vaccine antigens were as follows: (a) proteins with known function in *K. pneumoniae*, (b) essential cellular function, (c) surface exposure or secretion, (d) role in the pathogenicity and virulence of *K. pneumoniae*, and (e) complete sequence available to analyze their conservation at amino acid level among *K. pneumoniae* subsp. *pneumoniae* strains. Protein sequences were downloaded from the National Center for Biotechnology Information (NCBI) protein database (https://www.ncbi.nlm.nih.gov/protein/) (accessed 2025 May 5) and were analyzed as antigenic proteins using VaxiJen v2.0 tool (https://www.ddg-pharmfac.net/vaxijen/VaxiJen/VaxiJen.html) [87], with the default cutoff score established at 0.4 (previously employed for bacterial antigenic proteins [21,26,33,53,55,80]).

### Linear B cell epitope prediction and screening

Because B cell epitopes are essential in adaptive immunity triggering antibody-mediated responses, we established them as the key component for vaccine design. The linear B cell epitopes of the 8 target proteins were predicted using the BepiPred-3.0 server (https://services.healthtech.dtu.dk/services/BepiPred-3.0/) [88], the latest version that uses protein language models and outputs a score prediction per residue. The filters used for screening and selecting epitopes were as follows: (a) scores above the threshold by default (0.1512) for all proteins, except for siderophore receptors, for which a lower threshold was used (0.0802) because epitope prediction within this protein category did not reach the default value, thereby avoiding the exclusion of potentially relevant epitopes that otherwise failed to meet the predefined criteria, and (b) including the highest score values per residue, for siderophore receptors close to 0.12, and for the rest of the proteins to 0.2, which represented the maximum consensus scores achieved in the prediction. Table S1 includes all BepiPred-3.0 graphs showing linear B cell epitope predictions and the thresholds used as filters.

Due to variability in the length of B cell epitopes without adverse effects on the immune function [89,90], a consensus size of ~10 to 20 amino acids per epitope was established, favoring the selection of continuous epitopes that often adopt a structure similar to the native structure and promoting interactions with antibodies [91,92]. Given that the only predicted epitope of the Fiu protein meeting the previous selection criteria exceeded the consensus epitope length, a specific threshold of 0.12 was applied to bring it within the length range (Table S1).

The epitope screening process was validated using iBCE-EL (http://thegleelab.org/iBCE-EL/) [93], which predicts B cell epitopes considering the complete epitope sequence and its physicochemical properties. Furthermore, 2 additional tools, SEPPA 3.0 (http://www.badd-cao.net/seppa3/index.html) [94] and SEMA v2.0 (https://sema.airi.net/) [95], were used since these algorithms take into account the 3D structure of the sequence to predict epitopes. SEPPA 3.0 classifies the residues with an antigenicity score considering the subcellular localization of the antigen (secreted) and the species of the immune host (Homo) with an area under the curve (AUC) and balanced accuracy of 0.740 and 0.657, respectively. SEMA v2.0 outputs an epitope score based on the algorithm of the number of antibody residues in contact with any atom of antigen residues within a radius of 8 Å with AUC values of 0.744 and 0.777 for masked and unmasked epitope datasets, respectively.

A prior topology analysis was also performed for each candidate protein using the DeepTMHMM version 1.0.42 (https://dtu.biolib.com/DeepTMHMM) [96] and BOCTOPUS 2 (https://b2.topcons.net/) [97] tools to subsequently determine the localization (inside, outside, periplasm) and topology (α-helical or β-strand transmembrane, inner or outer loops) of the selected epitopes.

### Analysis of epitope sequence conservation

The 7 proteins in which antigens were found were used as queries in BLASTp tool to mine the database for sequences across different *K. pneumoniae* strains. To avoid partial sequences, a query coverage from 100% to 90% was established. Obtained sequences were manually inspected to eliminate those not from the target organism. Seven files, one per antigen, including between 316 and 1,141 protein sequences in FASTA format, were generated. The Epitope Conservancy Analysis, an analysis resource available in the IEDB server (http://tools.iedb.org/conservancy/) [98], was used to determine the percentage of conservation of all the epitopes used in each construct, setting the sequence identity threshold at 90% and 100%.

### Multi-epitope vaccine design

From the list of predicted B cell epitopes for each protein, 2 strategies were followed for the construction of multi-epitope vaccines by selecting one epitope per protein with (a) the highest residue score predicted by BepiPred-3.0 (BEPI-strategy) and (b) the highest antigenicity score predicted by VaxiJen v2.0 (VAXI-strategy). Our design criterion was to select one epitope per protein to compare constructs with the same number of epitopes and avoid biases toward one of the original proteins, due to length or favored by the different algorithms used by each program. This rationale to select one antigen per protein has been previously used by other authors [21,53,80].

To construct the multi-epitope vaccine, the selection of suitable amino acid linkers that ensure an effective *in vivo* separation of the joined single epitopes, allowing the preservation of the immunogenicity of each epitope, improving the epitope presentation to T and B cell receptors, and facilitating immune processing of vaccines *in vivo* [37,42,44,45,49], is required.

The 2 vaccine constructs were designed by assembling the chosen linear B cell epitopes with the use of KK peptide linkers, as supported by previous studies designing multi-epitope vaccines targeting *K. pneumoniae* [21,53,55]. Additionally, the Pan Dendritic-cell Receptor Epitope (PADRE), a widely used helper T cell stimulating epitope [50], was incorporated *in silico* into the multi-epitope sequences by using the GPGPG linker for its ability to induce helper T lymphocyte immune response [26,36,53,55]. At the C-terminal end of the constructs, a 6His-tag was added to aid in the purification of the vaccine proteins after expression in *E. coli* production systems [42,53,99].

### *In silico* codon optimization

To enable efficient expression of the vaccine constructs, the JCat server (https://www.jcat.de/) [100] was used for codon optimization to generate improved nucleotide sequences considering the codon adaptation index (CAI) and GC content values for favorable expression of foreign proteins depending on the intended expression host. We selected *E. coli* strain K12 as the expression organism in gene cloning experiments, and *H. sapiens* as the final target organism to use the designed vaccines. Additional parameters such as rho-independent transcription terminators [101,102] and prokaryotic ribosome binding sites [103] were avoided to ensure correct translation of the vaccine proteins. No restriction enzymes were selected. Because the algorithm of JCat is not perfectly suited for the human genome, optimization of the vaccine constructs was also performed using the Emboss backtranseq tool at EMBL-EBI platform (https://www.ebi.ac.uk/jdispatcher/st/emboss_backtranseq) [104] using the codon table of *H. sapiens*.

### Prediction of physicochemical properties and solubility analysis

Physicochemical properties of the designed vaccines, such as molecular weight (MW), isoelectric point (pI), instability index (II), aliphatic index (AI), grand average of hydropathicity (GRAVY), extinction coefficient, and *in vitro* and *in vivo* half-life, were assessed using Expasy ProtParam server (https://web.expasy.org/protparam/) [105]. Particular emphasis was placed on the II, as it was used to identify potentially unstable vaccine candidates. Other characteristics that were assessed were the degree of thermostability and the hydrophobic nature of the constructed vaccines. The estimation of the *in vivo* half-life of the potential vaccines was based on the principle of “N-end rule”, which states that amino acid nature of the extreme N-terminal affects the stability and half-life of the protein depending on the experimental model [106].

Vaccine solubility is a critical parameter for recombinant protein production and downstream processing. Two predictor tools were employed to assess solubility: SOLpro, available at Scratch protein prediction platform (https://scratch.proteomics.ics.uci.edu/) [107], and Protein-sol (https://protein-sol.manchester.ac.uk/) [108]. These algorithms assess solubility upon overexpression in *E. coli* with an overall accuracy of 74% and in an *E. coli*-based cell-free expression system with an accuracy of 58%, respectively. Furthermore, the SOLpro and Protein-sol algorithms have AUC values of 0.742 and 0.922 to 0.974, respectively, supporting their use as reliable classification models. An additional sequence-based solubility prediction was performed using the CamSol-Intrinsic v2.2 server (https://www-vendruscolo.ch.cam.ac.uk/camsolmethod.html) [109] for a deeper solubility analysis (per residue) and also taking into account generic aggregation propensity (pH value was set at the predicted theoretical pI). Considering that the 6His-tag may affect the overall solubility of the vaccine proteins [110,111], the analyses were performed using the 6His-tagged sequences.

### Multi-epitope vaccine construct secretion, virulence, and antigenicity profiling

SecretomeP-1.0 (https://services.healthtech.dtu.dk/services/SecretomeP-1.0/) [112] for mammals and SecretomeP-2.0 (https://services.healthtech.dtu.dk/services/SecretomeP-2.0/) [113] for Gram-negative bacteria were used to predict the secretory profile of the constructed vaccines. Both servers produce *ab initio* predictions of the non-classical secretion pathway, for example, proteins without an N-terminal signal peptide (like our vaccines). To study the potential virulence of the vaccine candidates, the VirulentPred2.0 tool (https://bioinfo.icgeb.res.in/virulent2/) [114], which outperforms other previous approaches considering accuracy, the latest virulent and non-virulent protein datasets, and the latest machine learning techniques, was employed. The antigenicity of the 2 multi-epitope vaccines was assessed using 2 tools, VaxiJen v2.0 (https://www.ddg-pharmfac.net/vaxijen/VaxiJen/VaxiJen.html) [87] and ANTIGENpro (https://scratch.proteomics.ics.uci.edu/) [115], which are alignment-independent antigen predictors based on the physicochemical properties or the protein sequences with different degrees of precision (70% to 89% or 76%), respectively.

### Safety of novel multi-epitope vaccines

Homology between multi-epitope vaccines and the human proteome was determined using the BLASTp module of the NCBI database (https://blast.ncbi.nlm.nih.gov/Blast.cgi?PROGRAM=blastp&PAGE_TYPE=BlastSearch&LINK_LOC=blasthome) [116,117], allergenicity by using AllerTOP v2.1 (https://www.ddg-pharmfac.net/allertop_test/) [118] and AllerCatPro v2.0 (https://allercatpro.bii.a-star.edu.sg/) [119] tools, and toxicity by using ToxDL (http://www.csbio.sjtu.edu.cn/bioinf/ToxDL/) [120] and ToxinPred2 (https://webs.iiitd.edu.in/raghava/toxinpred2/batch.html) [121] tools.

On the BLASTp server, the *H. sapiens* (taxid: 9,606) was selected as the target organism and the rest of the parameters were kept at default settings, including a threshold *E* value of 0.05.

For allergenicity analysis, AllerTOP v2.1 distinguishes between allergenic and non-allergenic proteins through the most probable route of exposure of tested proteins predicted as an allergen with an accuracy of 85.3%. In contrast, AllerCatPro v2.0 uses an extended dataset (including low allergenic proteins and autoimmune allergens), which improves the accuracy of allergenic potential compared to other computational methods.

The non-toxic nature of the vaccine constructs was evaluated using ToxDL tool that considers primary structure and domain embeddings for assessing protein toxicity, and ToxinPred2 tool that employs a hybrid approach and considers different physicochemical properties of the protein.

### Secondary structure prediction

To predict and characterize the secondary structure configurations of the generated multi-epitope vaccine constructs, the latest versions of PSIPRED V4.02 (http://bioinf.cs.ucl.ac.uk/psipred/) [122] and JPred4 (https://www.compbio.dundee.ac.uk/jpred4/index.html) [123] servers were used. PSIPRED V4.02 is one of the most accurate secondary structure generators with 84.2% accuracy by using deeper neural network architecture. Conversely, JPred4, based on the JNet v2.3.1 algorithm, predicts the secondary structure with an accuracy above 80%, the solvent accessibility of the protein structure including different degrees of accuracy that depends on the relative solvent accessibility thresholds used (>0%, >5%, and >25%), and the coiled-coil regions using the Lupas method [124]. As an output, the 2D features, including α-helix, β-sheet, and coil, were calculated showing the confidence score prediction. In the complementary analysis of solvent accessibility of the vaccine proteins, JPred4 represents the exposure or burial of each residue, with the corresponding reliability of prediction accuracy ranging from 0 to 9. Additionally, PSIPRED V4.02 also provides information on the types of amino acids (small non-polar, hydrophobic, polar, or aromatic) in the input sequence.

### Tertiary structure prediction, refinement, and validation

For 3D structural prediction, the Robetta server (https://robetta.bakerlab.org/) [125] was employed, which bases its modeling process on the *de novo* RosettaFold method when no match is found between the protein under study (our constructed vaccines) and a protein of known structure. This alternative method can be particularly suitable for *in silico* designed multi-epitope vaccine constructs [33,53,55,80] that result in artificial chimeric proteins containing flexible linkers and non-native combinations of peptide sequences. Furthermore, the resulting vaccine proteins do not necessarily adopt a single stable conformation comparable to that of naturally occurring proteins, which can pose challenge for other cutting-edge approaches, such as the AlphaFold algorithm, due to the absence of closely related experimental templates. To support the Robetta approach, we also conducted structural modeling using AlphaFold 3 (https://alphafoldserver.com/) [126]. The 5 predicted models estimated by Robetta were submitted into the GalaxyRefine server (https://galaxy.seoklab.org/cgi-bin/submit.cgi?type=REFINE) [127] to filter them and choose the optimized structure model among all the resulting approximations, considering the parameters global distance test—high accuracy (GDT-HA), root mean square deviation (RMSD), and MolProbity. Subsequently, the validation of the refined 3D protein structures of BEPI- and VAXI-vaccines was carried out using different tools. Firstly, we used the PROCHECK server [128], which determines the stereochemical quality of a protein structure by analyzing residue-by-residue geometry and overall structure geometry and outputs the Ramachandran plot where the relative proportion of amino acids that fall in the favored, allowed, and disallowed regions can be analyzed. Secondly, the VERIFY3D server [129] was used to determine the compatibility of an atomic model (3D) with its amino acid sequence (1D) by assigning a structural class based on its location and environment, and the ERRAT2 server [130] was used to analyze the error values based on the statistics of non-bonded interactions between different atom types. PROCHECK, VERIFY3D, and ERRAT2 tools are available at the structure validation server SAVES v6.1 (https://saves.mbi.ucla.edu/). Ultimately, ProSA server (https://prosa.services.came.sbg.ac.at/prosa.php) [131], a quality measure tool that uses experimentally solved protein structures in the Protein Data Bank (PDB) database, determined the overall 3D model quality score for BEPI and VAXI multi-epitope vaccine constructs. To visualize the 3D structures of the vaccine proteins, we employed Mol* 3D Viewer v5.0.0 (https://www.rcsb.org/3d-view) [132], a tool available at the Research Collaboratory for Structural Bioinformatics PDB (RCSB-PDB) platform.

### Analyses of the final multi-epitope vaccine 3D models

Further analyses of the 3D structure models may allow us to closely examine the behavior of the resulting vaccine proteins, as they are the final product of the constructed multi-epitope vaccines. In this regard, we first evaluated the accessible surface area by detecting exposed/buried residues protein with the Mol* 3D Viewer v5.0.0. Subsequently, the polarity of both vaccine proteins was determined by calculating the proportion of polar/non-polar residues with respect to the total residues exposed. In addition, structure-based solubility predictions were performed to assess the degree of solubility of the vaccine conformational proteins. We used the CamSol-Structurally corrected v2.3 server (https://www-vendruscolo.ch.cam.ac.uk/camsolmethod.html) [109], which calculates a solubility profile accounting for the proximity of the amino acids in the structure and for their solvent exposure, and the SOLart v1.0 tool (http://babylone.ulb.ac.be/SOLART/) [133], whose algorithm works with the backbone torsion angle potential, the solvent accessibility potential, and the inter-residue distance potential using datasets of *E. coli* proteins with experimental structures and solubility values.

### *In silico* immune response simulation

Prediction of the potential immune response elicited upon administration of the generated vaccine constructs was performed using the C-ImmSim server (https://kraken.iac.rm.cnr.it/C-IMMSIM/index.php) [134]. This server works with recombinant protein vaccines and simultaneously stimulates 3 separate anatomical regions in mammals, including the bone marrow, the thymus, and a tertiary lymphatic organ (a lymph node). It uses position specific scoring matrix (PSSM)-based method and machine learning approaches to predict the peptides and immunological interactions, outputting detailed humoral and cellular responses as well as specific immuno-molecular components.

Simulated vaccination with the 2 multi-epitope vaccines consisted of 3 doses [21,26,33,53,55,80] administered every 2 weeks (injection time-steps were 3, 42, and 84), resulting in 540 simulation steps (each time-step equals 8 h in real life, and time-step 3 is injection at time 0 with the first dose). Time-steps (dose intervals) vary between immunoinformatics studies [26,33,39,45,46,80,82], so we decided to follow the *in vivo* immunization protocol based on previous validated experimental immunization schedules used in our laboratory. The volume and number of antigens to inject were 10 and 1,000, respectively [21,26,33,53,80]. The vaccine did not include LPS, but a default quantity of adjuvant was necessary. Random seed was arbitrarily set at 12,000, a similar value used in other previous works [21,33,53,80]. Finally, host human leukocyte antigen (HLA) types used were left by default, being A0101 and B0702 for major histocompatibility complex class I (MHC-I), and DRB1_0101 for MHC-II.

### Population coverage analysis

Although our vaccine constructs were primarily based on predicted linear B cell epitopes, population coverage analysis was performed to estimate the theoretical capacity of the selected peptide regions to be presented by diverse HLA alleles following antigen processing. A similar methodology was previously used to prioritize B cell-derived MHC-I and MHC-II epitopes in a multi-epitope vaccine design [83]. Other approaches have demonstrated the existence of overlapping B cell and T cell epitopes [29,53]. To conduct our comparative *in silico* study, we performed a population coverage analysis for both multi-epitope vaccines using the IEDB v3.0.2 server (https://tools.iedb.org/population/) [135] and selecting worldwide and 16 geographical areas (by default) for class I separate, class II separate, and class I and class II combined calculations. We previously evaluated the HLA allele binding predictions to select HLA class I and class II alleles that bind to our vaccine B cell epitopes. For this analysis, we also used the IEDB server, specifically the MHC-I Binding Predictions v3.1.6 (https://tools.iedb.org/mhci/) and MHC-II Binding Predictions v3.1.12 (https://tools.iedb.org/mhcii/) tools based on the recommended binding predictors NetMHCpan-4.1 BA and NetMHCIIpan-4.0 BA, respectively [136]. We employed the full reference sets of HLA class I and class II alleles that provide more than 97% [137] and 99% [138] population coverage, respectively, and the rest of the parameters were kept at default settings. HLA alleles whose binding prediction, calculated as median inhibitory concentration (IC_50_) value, was <500 nM were selected for population coverage analysis for each multi-epitope vaccine, as high and intermediate binding affinity to a peptide was included (<50 and <500 nM, respectively) (Table S3). The IC_50_ value <500 nM is an established threshold associated with immunogenicity [139].

### Data validation

To optimize the overall accuracy of the methodology and obtain consistent results, 2 or more bioinformatics tools or approaches were used for each analysis in addition to selecting the latest or improved tools at every stage of the workflow. To create a more reliable model of the designed vaccines, an integrated approach combining physicochemical properties based on sequence and structure methods and the prediction of secondary and 3D structures was used. Finally, to conclude the study, the immunogenicity experiment was simulated and a worldwide population coverage analysis was performed. The software version, parameter settings, and input file required for each bioinformatics or immunoinformatics analysis are detailed in Table S3.

## Results

### Selection of candidate proteins for multi-epitope vaccine construction

MrkA, OmpA, OmpW, LamB, PhoE, FepA, FhuA, and Fiu were the target proteins for our study. The 8 proteins were filtered using their antigenicity score calculated by VaxiJen v2.0. The antigenic score of each protein, its function, its length, and its accession number are detailed in Table 1. The highest probabilities for inducing an immune response were predicted for MrkA, LamB, PhoE, and FepA, representatives of each functional group of proteins.

**Table 1.** List of *Klebsiella pneumoniae* proteins selected as potential antigens. Accession numbers were obtained from the NCBI protein database, and protein antigenicity was verified using VaxiJen v2.0 tool.

Protein group	Protein name	Description and function	Accession number	Length (amino acids)	Antigenicity score ^a^
Group I Fimbriae	MrkA	Major and structural subunit of type 3 fimbriae Biofilm formation, adherence to abiotic surfaces, and host cell attachment	QPW33900.1	202	0.79
Group II Outer membrane proteins and porins	OmpA	Major outer membrane protein of Gram-negative bacteria Maintenance of structural cell integrity, role in bacterial conjugation, bacteriophage binding and invasion of mammalian cells, and cell growth	QPW36129.1	356	0.65
	OmpW	Small outer membrane protein Transport of small hydrophobic molecules, protection against environmental stress, and receptor for colicin S4	QPW35866.1	212	0.75
	LamB	Maltoporin, trimeric integral outer membrane protein Transport of maltose and maltodextrins, and relation with antimicrobial resistance	QPW32826.1	429	0.77
	PhoE	Phosphorin, trimeric integral outer membrane protein Specific transport of anions but no for phosphates, stabilization and fluidization of the outer membrane, and relation with antimicrobial resistance	QPW32145.1	350	0.77
Group III Siderophore receptors	FepA	Ferrienterobactin siderophore receptor Uptake of iron complexed with the ferrienterobactin siderophore, and receptor for colicin B and D Infection and colonization stages	AEW58984.1	752	0.79
	FhuA	Ferrichrome siderophore receptor Uptake of iron in complex with ferrichrome siderophore (ferric hydroxamate transport) Infection and colonization stages	QPW32251.1	735	0.60
	Fiu	Catecholate siderophore receptor Transport of iron-catecholate complexes Infection and colonization stages	QPW35864.1	761	0.71

^a^ Cutoff of the selection criteria of the immunoinformatics tool used for prediction: >0.4.

### B cell epitope prediction and selection

The B cell epitopes for each protein were predicted using immunoinformatics tools, and the final peptides of interest to construct our multi-epitope vaccines are listed in Table 2. For all proteins, except FhuA and Fiu, 2 different B cell epitopes were predicted by each approach; thus, 2 different vaccines were constructed. At this step, the FhuA protein was excluded as a candidate because its only predicted peptide was not above the antigenicity threshold used, which was in concordance with the lowest antigenicity score as a native protein (0.60). Following the BEPI-strategy, the fimbrial subunit and porins contained the residues with the highest scores (0.21 to 0.25), and the lowest values corresponded to the siderophore receptors (0.12 to 0.16). Regarding the VAXI-strategy, the predicted antigenicity scores were greater than 1.0 for all epitopes, with the most antigenic being 2.49 found in the PhoE protein. The peptides chosen from the 7 proteins in this study were successfully validated as B cell epitopes, considering epitope sequence (probability value >0.35), epitope antigenicity (average antigenicity score >0.089), and epitope structure (average epitope score >0.361) (Table 2). When considering 3D structures, SEPPA 3.0 classified almost all residues as antigenic (except for 15/156 from the BEPI-vaccine and 11/124 from the VAXI-vaccine that scored below the threshold of 0.089), and SEMA v2.0 predicted 92% of the BEPI-vaccine and 94% of the VAXI-vaccine as epitope regions (143/156 and 117/124 residues scored above the threshold of 0.361, respectively) (data not shown). Interestingly, the residue scores for the Fiu epitope differed between the 2 vaccine constructs (data not shown), indicating the distinct conformation adopted by the same epitope when the global conformational structure of the protein was considered. This highlights the importance of also applying these 3D approaches to the resulting multi-epitope vaccine constructs. Furthermore, almost all predicted peptides were localized to external regions of the corresponding native proteins (average probability value ≥0.91) and were topologically predicted as loops (average probability value ≥0.93) (Table 3). Only one epitope of OmpA and 2 from MrkA were the exceptions (inner loops). The external localization and internal loops of the MrkA epitopes can be explained by the polymerization of MrkA subunits from inside to outside to form type 3 fimbriae [56,60]. Antigen conservation analysis evidenced a variable level of conservation of the selected epitopes depending on the antigenic protein and strategy used. In general, conservancy values for the epitopes selected for the VAXI-strategy (81.07% to 96.31%) were higher than those for the epitopes included in the BEPI-strategy (76.12% to 91.88%) at 100% sequence identity threshold and conservancy was above 94% for all epitopes when establishing the threshold at 90% (Table 2). Table S1 shows the list of all B cell epitopes predicted for each protein using the BepiPred-3.0 tool.

**Table 2.** List of B cell epitopes selected for the construction of BEPI and VAXI multi-epitope vaccines. The BepiPred-3.0 and VaxiJen v2.0 scores marked in bold are the selected ones depending on the criterion followed for the multi-epitope vaccine construction.

			Linear B cell epitope prediction		Criteria		Validation			Conservancy	
			BepiPred-3.0		BepiPred-3.0	VaxiJen v2.0	iBCE-EL	SEPPA 3.0	SEMA 2.0	IEDB	
Protein (family)	Construct	Epitope name	Peptide	Length	Highest score residue (BEPI) ^a^	Highest antigenicity score (VAXI) ^b^	BCE prob value ^c^	Ave ag score ^d^	Ave ep score ^e^	% seq 90% similar (no. seq/total seq)	% seq 100% similar (no. seq/total seq)
MrkA (Fim)	BEPI	MrkA-B	GNLLAGATSKQQGYLANTEASGA	23	**0.251381**	0.70	0.96	0.848	0.503	96.12% (322/335)	76.12% (255/335)
	VAXI	MrkA-V	CQAADGTKQDDVS	13	0.243554	**1.80**	0.79	0.220	0.666	94.38% (319/338)	81.07% (274/338)
OmpA (OMP)	BEPI	OmpA-B	DTGFYGNGFQNNNGPTRN	18	**0.240455**	0.49	0.96	0.572	0.464	93.78% (407/434)	85.48% (371/434)
	VAXI	OmpA-V	NTCDNVKARAALI	13	0.226775	**1.07**	0.63	0.118	0.437	99.77% (433/434)	96.31% (418/434)
OmpW (OMP)	BEPI	OmpW-B	TEGSDNVLGSLGSFNVS	17	**0.218183**	0.74	0.96	0.118	0.423	99.68% (315/316)	89.24% (282/316)
	VAXI	OmpW-V	KFKAGGVDQKVST	13	0.202063	**1.81**	0.96	0.104	0.630	98.73% (312/316)	88.61% (280/316)
LamB (Por)	BEPI	LamB-B	SGGSYTFSSDDTKKYAA	17	**0.246753**	0.96	0.96	0.125	0.399	95.13% (820/862)	83.18% (717/862)
	VAXI	LamB-V	PQDDYRLEDGA	11	0.224832	**1.22**	0.82	0.147	0.580	99.54% (858/862)	91.53% (789/862)
PhoE (Por)	BEPI	PhoE-B	LKSDNKLGIND	11	**0.240285**	1.13	0.96	0.191	0.450	94.96% (339/357)	91.88% (328/357)
	VAXI	PhoE-V	SDYDSKDGD	9	0.205213	**2.49**	0.96	0.185	0.652	94.12% (336/357)	94.12% (336/357)
FepA (SidR)	BEPI	FepA-B	SRMDEGLAGGTEGIFSSS	18	**0.122280**	0.75	0.96	0.100	0.433	96.74% (386/399)	81.20% (324/399)
	VAXI	FepA-V	KRFNYKGEAVSGS	13	0.117577	**1.45**	0.96	0.164	0.434	97.74% (390/399)	87.72% (350/399)
Fiu (SidR)	BEPI and VAXI	Fiu-B/V	CGGSGRGAIACPPGQSTGSPV	21	**0.165908**	**1.24**	0.96	0.121 0.144	0.706 0.553	100.00% (1,141/1,141)	94.57% (1,079/1,141)

Fim, fimbriae; OMP, outer membrane protein; Por, porin; SidR, siderophore receptor; BCE, B cell epitope; prob, probability; ave, average; ag, antigenicity; ep, epitope; seq, sequences; no, number

^a^ Cutoff of the selection criteria of the immunoinformatics tools used for predictions: >0.2 for Fim, OMP, and Por, and >0.12 for SidR.

^b^ Cutoff of the selection criteria of the immunoinformatics tools used for predictions: >0.4.

^c^ Cutoff of the selection criteria of the immunoinformatics tools used for predictions: >0.35.

^d^ Cutoff of the selection criteria of the immunoinformatics tools used for predictions: >0.089.

^e^ Cutoff of the selection criteria of the immunoinformatics tools used for predictions: >0.361.

**Table 3.** Position, localization, and topology of the selected predicted epitopes in the original native proteins using BepiPred-3.0, DeepTMHMM v1.0.42, and BOCTOPUS 2 tools.

	Epitope position		Epitope localization	Epitope topology
Epitope name	Start	End	DeepTMHMM v1.0.42	BOCTOPUS 2
			Average probability	Average probability
MrkA-B	108	130	Outside (0.99)	Inner loop (1.0)
MrkA-V	87	99	Outside (0.99)	Inner loop (1.0)
OmpA-B	41	58	Outside (1.0)	Outer loop (0.99)
OmpA-V	319	331	Periplasm (1.0)	Inner loop (1.0)
OmpW-B	39	55	Outside (0.95)	Outer loop (0.99)
OmpW-V	185	197	Outside (0.99)	Outer loop (0.99)
LamB-B	174	190	Outside (1.0)	Outer loop (0.99)
LamB-V	223	233	Outside (1.0)	Outer loop (0.93)
PhoE-B	328	338	Outside (0.97)	Outer loop (0.99)
PhoE-V	45	53	Outside (0.91)	Outer loop (0.96)
FepA-B	349	366	Outside (0.99)	Outer loop (0.99)
FepA-V	662	674	Outside (0.99)	Outer loop (0.99)
Fiu-B/V	482	502	Outside (1.0)	Outer loop (0.99)

### Construction of the BEPI and VAXI multi-epitope vaccines

At the vaccine construction stage, we linked the final 7 B cell epitopes together using KK linkers. The PADRE peptide was fused to the multi-epitope sequences with the GPGPG linker to enhance CD4^+^ T cell responses and potentiate the immune response generated by the designed vaccines. Following that, 6 histidines were incorporated at the end of the constructs as a tag for vaccine purification. The resulting BEPI- and VAXI-constructs constituted 156 and 124 amino acids, respectively, and their complete sequences are represented in Fig. 2 (also included as Textfile S1).

**Fig. 2.**
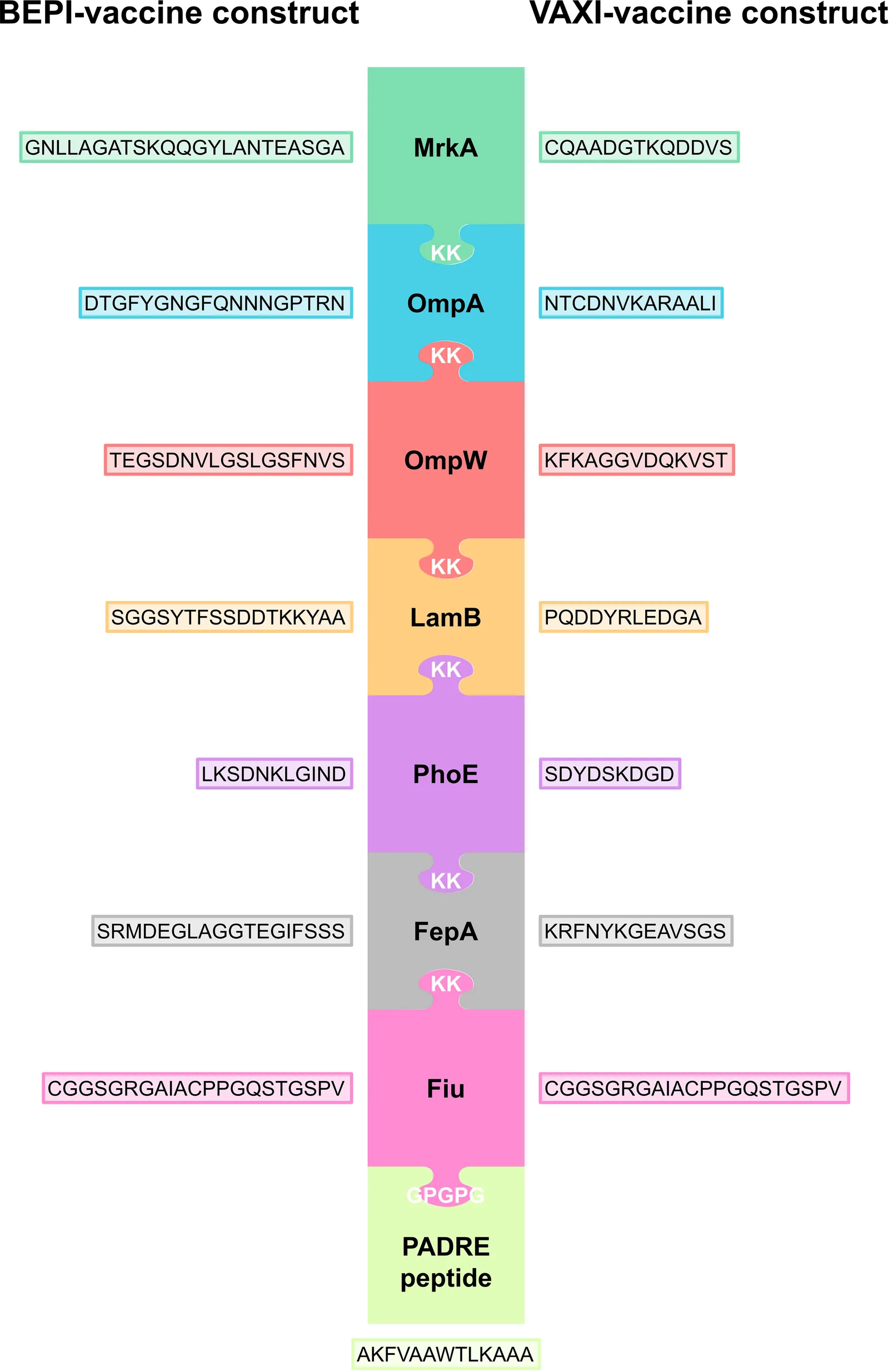
Schematic representation of the multi-epitope vaccine constructs. BEPI and VAXI multi-epitope vaccines were constructed with one B cell epitope from each virulence factor and the PADRE peptide. KK and GPGPG represent linkers. The methionine amino acid (as start codon) and the 6His-tag at the extreme C terminus are not included in the figure.

### Codon optimization of the BEPI and VAXI multi-epitope sequences

The 2 multi-epitope construct sequences were optimized for expression in *E. coli* and *H. sapiens* (Textfile S2). Analysis with the JCat server showed a CAI superior to 0.95 in *E. coli* K-12 and *H. sapiens* (1.0 and 0.96 for the BEPI-vaccine construct, and 0.96 and 0.96 for the VAXI-vaccine construct, respectively), which indicated a favorable predicted expression in both organisms (the accepted range is between 0.8 and 1.0). In addition, the calculated GC content of both vaccine sequences adapted to *E. coli* K-12 was around 50% (49.4% and 50.5% for BEPI- and VAXI-constructs, respectively), while the value for adaptation to *H. sapiens* was >68% (68.2% and 68.8% for BEPI- and VAXI-constructs, respectively), very close to the limit of the accepted range, which is between 30% and 70%. The results suggest that both optimized sequences would likely be efficiently transcribed and translated.

### Physicochemical characteristics

The constructed vaccine candidates were analyzed physically and chemically to gain insights into their nature, behavior, and composition. Both vaccines were predicted to be stable, with an II around 27 (a value above 40 predicts that the protein may be unstable) and an AI, which measures the thermostability, ranging between 45 and 50. Hydrophilicity of the designed vaccine constructs was determined by their GRAVY index, which was negative in both cases (for the BEPI-vaccine was −0.70 and for the VAXI-vaccine was −0.92), indicating that vaccine proteins were predicted to be hydrophilic. This prediction would be supported by the higher content of polar amino acids in comparison with hydrophobic amino acids (31% *vs*. 12% in the BEPI-vaccine construct, and 39% *vs*. 10% in the VAXI-vaccine construct, respectively) (Fig. S1A and B). Furthermore, they were predicted to behave like basic proteins, with a pI around 9.7. The estimated half-life results were the same for both vaccines in the 3 model organisms analyzed (mammals, yeast, and *E. coli*). The longest estimated half-life was found in mammalian reticulocytes *in vitro* with a time prediction of 30 h inside cells after being synthesized. *In vivo* half-life predictions in yeast and *E. coli* were of >20 h and >10 h, respectively. Other parameters included an MW of approximately 16 and 13 kDa for BEPI- and VAXI-vaccines, respectively, and a similar extinction coefficient between both vaccine candidates (Table 4).

**Table 4.** Physicochemical properties of the final multi-epitope vaccine constructs using Expasy ProtParam server and solubility analysis using SOLpro, Protein-sol, and CamSol v2.2 tools. The best predicted scores for the physicochemical characteristics between both vaccine constructs are boldfaced.

Parameters		BEPI-vaccine construct	VAXI-vaccine construct
Number of amino acids		156	124
Molecular weight		15,795.54 Da	**12,927.62 Da**
Theoretical isoelectric point		9.75	9.65
Instability index ^a^		27.29	**26.33**
Aliphatic index ^b^		47.69	45.00
Grand average of hydropathicity		−0.690	**−0.915**
Extinction coefficient		**11,585 M**^**−1**^ **cm**^**−1**^	10,220 M^−1^ cm^−1^
Estimated half-life	Mammalian reticulocytes *in vitro*	30 h	30 h
	Yeast *in vivo*	>20 h	>20 h
	*Escherichia coli in vivo*	>10 h	>10 h
Solubility	Upon overexpression ^c^	**0.87**	0.80
	Cell-free expression system ^d^	0.78	**0.80**
	Intrinsic solubility ^e^	2.10	**2.49**

^a^ Cutoff of the selection criteria of the immunoinformatics tools used for predictions: <40.

^b^ Cutoff of the selection criteria of the immunoinformatics tools used for predictions: 45 to 50.

^c^ Cutoff of the selection criteria of the immunoinformatics tools used for predictions: >0.5.

^d^ Cutoff of the selection criteria of the immunoinformatics tools used for predictions: >0.45.

^e^ Cutoff of the selection criteria of the immunoinformatics tools used for predictions: >1.

The vaccine designs were also analyzed for their solubility upon overexpression in *E. coli* using the SOLpro server and obtained the positive values of 0.87 for the BEPI-vaccine and 0.80 for the VAXI-vaccine (a score that exceeds 0.5 is positive for solubility). Another complementary approach for solubility prediction was the use of the Protein-sol tool, where any scaled solubility value greater than 0.45 is predicted to have a higher solubility than the average soluble *E. coli* protein from the experimental solubility dataset. In this line, both vaccines were also predicted to be soluble, with scores of 0.78 and 0.80 for BEPI- and VAXI-vaccines, respectively. Protein-sol also provides additional information on the contribution of different characteristics to solubility, identification of net charge subdomains, and detection of folding subdomains of the 2 vaccine proteins (Fig. S2A and B). According to the analysis of solubility or aggregation propensity per residue, both vaccines proteins were also predicted to be soluble with scores >1 (2.10 for the BEPI-vaccine and 2.48 for the VAXI-vaccine). CamSol-Intrinsic v2.2 tool classifies the residues as highly soluble (>1) or poorly soluble (<−1) and provides the intrinsic solubility profile (Fig. S7A and C). All physicochemical parameters studied for the 2 multi-epitope constructs are included in Table 4.

### Secretion, virulence, and antigenicity profile of the BEPI- and VAXI-vaccines

Once the physicochemical profile was analyzed, the secretion, virulence, and antigenicity characteristics of both vaccines were assessed (Table 5).

**Table 5.** Secretion, virulence, antigenicity, and potential safety of the BEPI and VAXI multi-epitope vaccines. The best predicted scores for each characteristic between both vaccine constructs are boldfaced.

Vaccine characteristic	Tool	BEPI-vaccine construct	VAXI-vaccine construct
Secretion	SecretomeP-2.0 ^a^ Gram-negative bacteria	**Secretory protein (SecP score: 0.87)**	Secretory protein (SecP score: 0.75)
	SecretomeP-1.0 ^b^ Mammalian	**Secretory protein (NN score: 0.91)**	Secretory protein (NN score: 0.87)
Virulence	VirulentPred 2.0 ^c^	Virulent protein	Virulent protein
Antigenicity	VaxiJen v2.0 ^d^	0.91	**1.37**
	ANTIGENpro ^e^	**0.85**	0.66
Human homology	BLASTp ^f^	Non-homologous	Non-homologous
Allergenicity	AllerTOP v2.1 ^g^	Non-allergen	Non-allergen
	AllerCatPro v2.0 ^h^	No evidence (no hits)	No evidence (no hits)
Toxicity	ToxDL	**Non-toxic domains (Toxicity score: 0.0012)**	Non-toxic domains (Toxicity score: 0.10)
	ToxinPred2 ^i^	**Non-toxin (Hybrid score: 0.22)**	Non-toxin (Hybrid score: 0.41)

^a^ Cutoff of the selection criteria of the immunoinformatics tools used for predictions: >0.5.

^b^ Cutoff of the selection criteria of the immunoinformatics tools used for predictions: >0.6.

^c^ Cutoff of the selection criteria of the immunoinformatics tools used for predictions: >0.5.

^d^ Cutoff of the selection criteria of the immunoinformatics tools used for predictions: >0.4.

^e^ Cutoff of the selection criteria of the immunoinformatics tools used for predictions: >0.5.

^f^ *E* value 0.05.

^g^ Cutoff of the selection criteria of the immunoinformatics tools used for predictions: >0.5.

^h^ A 93% identity of 3D epitopes, a 35% identity over 80 linear residues, and 3 × hexamers.

^i^ Cutoff of the selection criteria of the immunoinformatics tools used for predictions: 0.6.

Firstly, BEPI- and VAXI-protein vaccines were predicted to be secreted into the bacterial expression system as their SecP scores were 0.87 and 0.75, respectively (the recommended threshold is 0.5 in Gram-negative bacteria sequences). In parallel, both multi-epitope sequences were predicted to produce mammalian secretory proteins as well, with NN scores of 0.91 for the BEPI-vaccine and 0.87 for the VAXI-vaccine (scores above 0.6 in mammals are considered indicative of secretion).

Secondly, the resulting multi-epitope products should constitute virulence-associated proteins to simulate an infection by *K. pneumoniae*. As both constructs were above 0.5, BEPI- and VAXI-vaccines were considered virulence-associated.

The potential for antigenicity of the final designed vaccine constructs was also analyzed. The VAXI-vaccine scored higher than the BEPI-vaccine (1.37 *vs*. 0.91) by VaxiJen v2.0, while the antigenicity predictor ANTIGENpro estimated the BEPI-vaccine as more antigenic (score of 0.85) compared to the VAXI-vaccine (score of 0.66). In conclusion, the antigenicity of the BEPI and VAXI whole vaccine constructs was above the threshold of 0.4, and also higher than the original protein scores, suggesting an enhanced predicted antigenic potential and that they would likely act as better protective antigens.

### Human homology, allergenicity, and toxicity of the designed vaccines

Prior to the secondary and tertiary structure predictions of the potential vaccines created *in silico*, it was essential to assess their predicted safety with respect to non-similarity to human proteome, non-allergenicity, and non-toxicity. There was no similarity (0%) between the vaccine constructs’ query coverage and the proteins of *H. sapiens*, based on the sequence homology between the generated vaccine proteins and the human proteome database (Table 5).

The allergenicity and toxicity servers’ outputs revealed that both potential vaccines were predicted to be probable non-allergens (classification based on the most similar protein and with no evidence of similarity to allergens) and non-toxic proteins (with a toxicity score below the threshold of 0.6 and no toxic domains detected) (Table 5). Therefore, BEPI- and VAXI-vaccines were predicted not to cause autoimmune responses in the host due to their similarity to human proteins or allergens, nor would they cause any toxic reaction in the body.

### Secondary structure and solvent accessibility analysis

The secondary structures of the designed vaccines were evaluated. The PSIPRED V4.02 prediction results showed that the BEPI-vaccine would have 17% α-helix domains, 12% β-strand regions, and 71% random coils (Fig. 3A), and the VAXI-vaccine would have 16% α-helix, 8% β-strand, and 76% random coils (Fig. 3B). The secondary structural domain proportions for both vaccines analyzed by JPred4 were also similar (Fig. S3A and B). As expected, most regions corresponded to random coils, suggesting that the vaccine configurations would expose epitopes on the surface promoting epitope accessibility and antibody recognition [53,140]. This assumption was supported by the fact that all epitopes selected for BEPI- and VAXI-vaccine constructs were predicted to be loops in their corresponding native proteins. Furthermore, the high number of α-helices, which would help in the maintenance of the stability of the vaccine protein structures, together with a high proportion of coiled-coil regions, would indicate a correct protein folding, helping to maintain the native protein structures [26,36,37,53,140,141]. Therefore, it can be proposed that BEPI- and VAXI-vaccines would constitute potential structural antigens facilitating antibody recognition and allowing the development of a functional humoral immunity against the target pathogen. This presumption may be supported by the solvent accessibility result. Considering the threshold of 5%, which means that the residues with B are 95% buried, we observed that BEPI- and VAXI-vaccines would have a conformation with almost all regions exposed according to their secondary structure predictions (Fig. S3A and B).

**Fig. 3.**
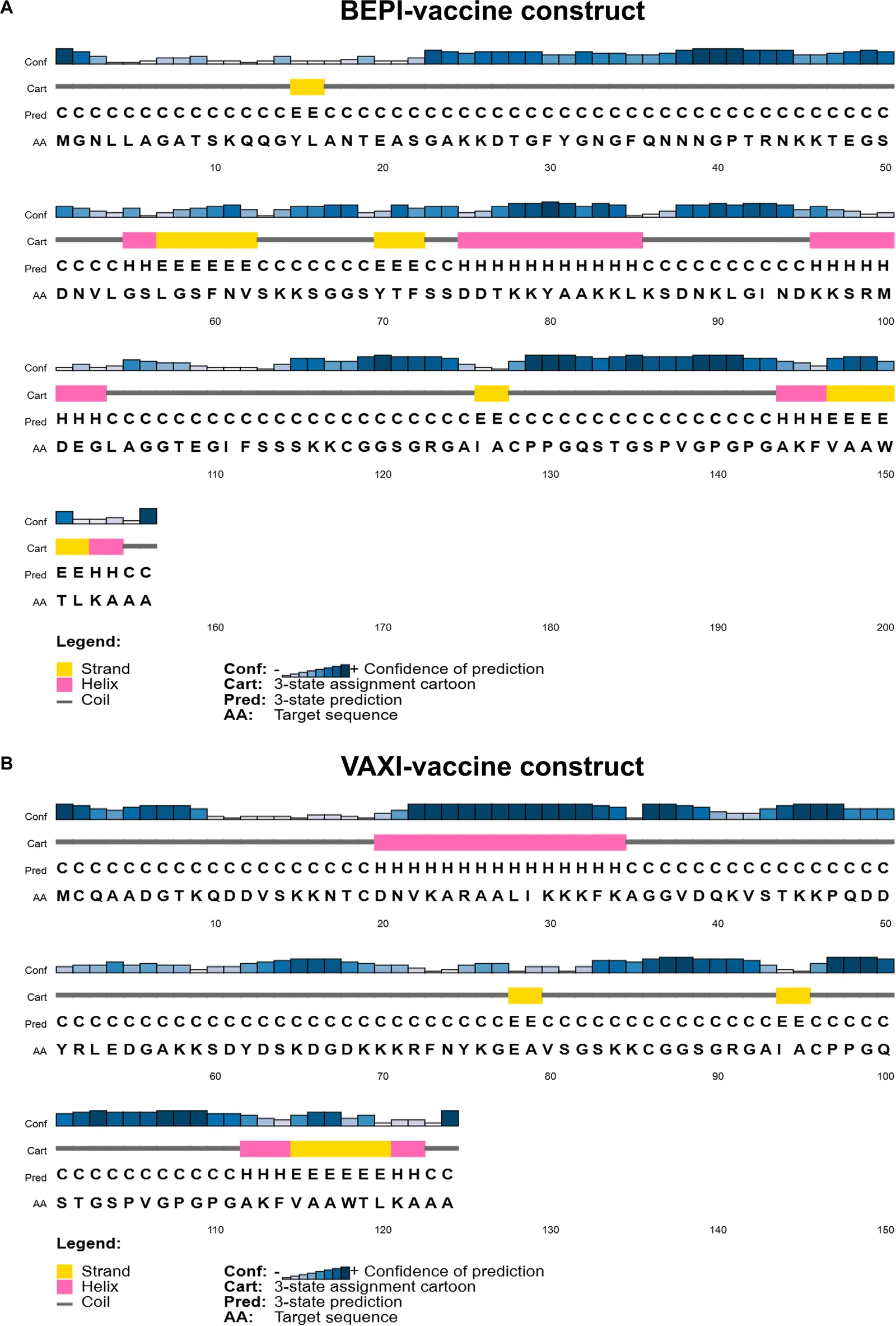
Secondary structure prediction of the final multi-epitope vaccine constructs using PSIPRED V4.02 tool. (A) BEPI-vaccine construct. (B) VAXI-vaccine construct.

### Determination and validation of the 3D structures of the BEPI and VAXI multi-epitope vaccines

The 3D structures of the final multi-epitope vaccine constructs were generated using different tools for modeling. From Robetta prediction, with a confidence of 0.40 for the BEPI-construct and 0.48 for the VAXI-construct, model 5 was selected for refinement. According to the AlphaFold 3 prediction, model 0 was the best predicted, considering the ranking score (0.63/0.74), the predicted template modeling score (pTM) (0.21/0.24), and the disordered fraction (0.84/0.99) for the BEPI- and VAXI-constructs, respectively. AlphaFold also generates a per-residue confidence score (pLDDT) between 0 and 100, and some regions with low pLDDT may be unstructured in isolation [142]. In addition to low confidence levels in the overall structure (pTM) and high disordered fractions (predetermined ranges of 0 to 1), the predicted BEPI- and VAXI-vaccine models exhibited tertiary structures in which the majority of regions appeared as unresolved loops with low (70 > pLDDT > 50) and very low scores (pLDDT < 50) (Fig. S4A and B). Consequently, the 3D structure models predicted by Robetta were selected for further analyses. GalaxyRefine output 5 refined models that were presented with GDT-HA, RMSD, and MolProbity scores describing the structural changes after optimization compared to the initial Robetta approximation. GDT-HA is the high accuracy of the global distance score, ranging from 0 to 1, with a higher value indicating better model accuracy. RMSD measures the root mean square deviation of Cα atom positioning in comparison to the native structure, being a lower RMSD favorable. MolProbity combines clash, poor rotamers, and favored Ramachandran scores to assess the quality of all model atoms and their bonds, with a lower score being ideal [127]. According to this, model number 5 for the BEPI-construct and model number 3 for the VAXI-construct displayed the best characteristics (GDT-HA: 0.9808/0.9960, RMSD: 0.355/0.275, MolProbity: 1.612 Å/1.772 Å, clash score: 9.3/6.5, poor rotamers: 0.9/1.1, and Rama favored: 97.4/94.3, respectively) and were chosen as the final structures (Fig. 4A and B) for further analysis. GDT-HA values close to 1 indicated that the refined BEPI- and VAXI-structures would not deviate greatly from the initial models (with a default value of 1). In the case of the refined BEPI-model, the Ramachandran value of 97.4 was an improvement over the original structure, which scored 95.5, meaning that at least 97.4 of the residues were predicted to be in the most stable conformational zones. In agreement, the Ramachandran plots calculated by PROCHECK (Fig. 4C and D) after model refinement showed that maximum residues fell in the most favored regions in the BEPI- and VAXI-structures (93.4%/86%), followed by the percentage of residues in allowed regions including additional and generously allowed regions (6.6%/13.0%), and without any residue or only one residue in disallowed regions, respectively. These positive results suggested that both vaccines would generate a 3D configuration in an ideal state with stereochemical stability. Furthermore, the overall quality and potential error of the final structures was determined by ProSA, providing a *Z* score of −4.1, which revealed that resulting modeled proteins were predicted to be present within the range of x-ray solved protein structures with the same sequence length as demonstrated in Fig. 4E and F, indicating that BEPI- and VAXI-structures would contain no errors. Complementary analysis of local model quality by ProSA also confirmed the high quality and the successful prediction of the tertiary structures (data not shown).

**Fig. 4.**
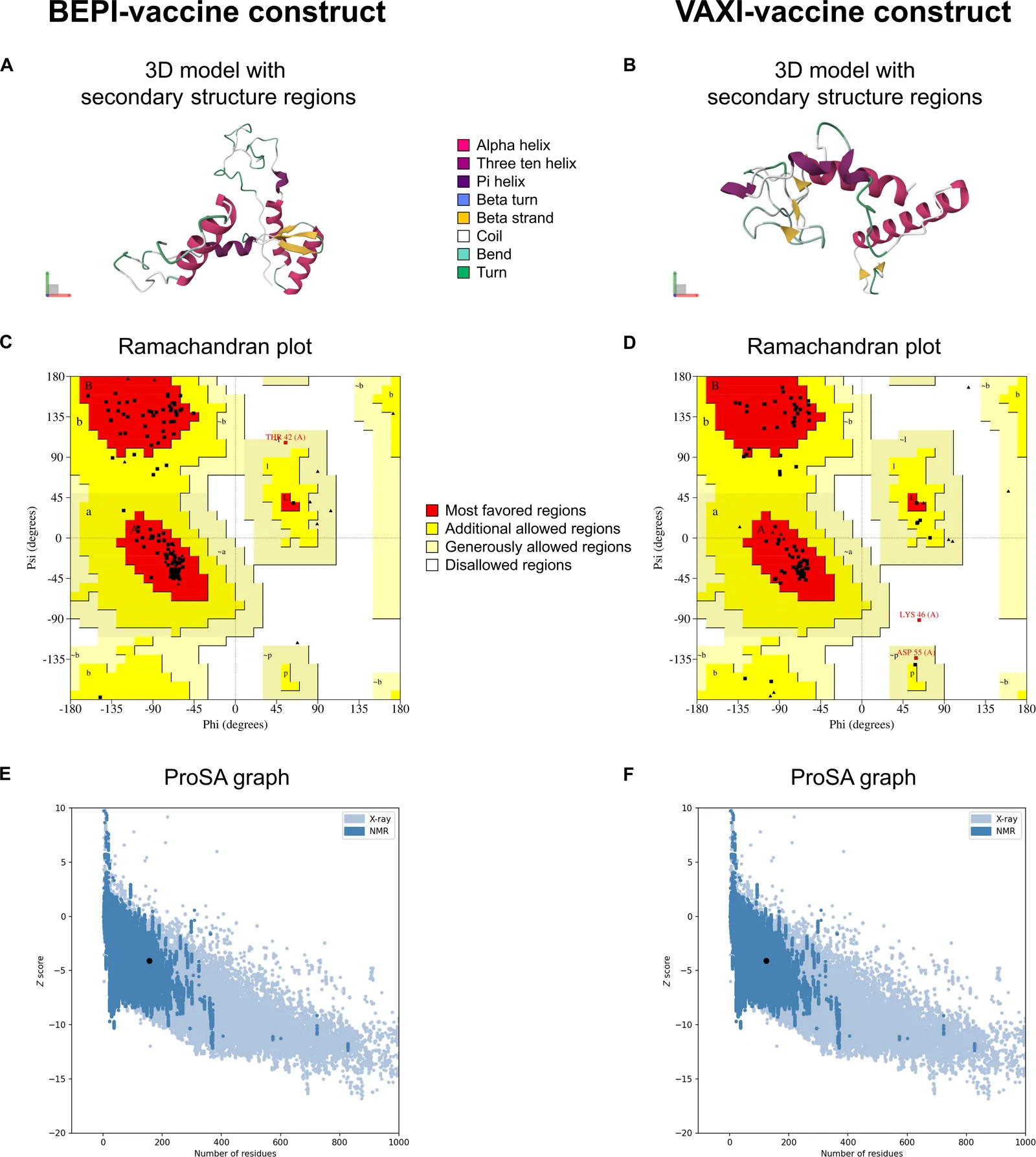
Structural analysis of the multi-epitope vaccine tertiary structures using Robetta, GalaxyRefine, Mol* 3D Viewer v5.0.0, PROCHECK (SAVES v6.1), and ProSA tools. (A and B) Visualization of the 3D models labeled with secondary structure regions. (C and D) Ramachandran plots showing residues in favored or disallowed regions. (E and F) ProSA graphs including the *Z* score (black dot). All structural analyses for BEPI- and VAXI-vaccine constructs were performed after refinement.

Additional results for structure validation are shown in Fig. S5. High scoring values above 50% for BEPI- and VAXI-structures (90.84%/85.42%, respectively) were obtained by ERRAT2 analysis (Fig. S5A and B). Both vaccine structural models also passed the VERIFY3D analysis, showing that at least 80% of the amino acids scored ≥0.1 in the 3D/1D profile (91.03% for the BEPI-vaccine and 83.06% for the VAXI-vaccine) (Fig. S5C and D). Conclusively, the 3D structures of the 2 multi-epitope vaccines developed were accurately and reliably predicted.

### Accessible surface area, polarity, and solubility of the 3D multi-epitope vaccine models

The final 3D structure models (after refinement) were used to determine the accessible surface area of both vaccine proteins. The exposed and buried protein residues are shown in Fig. S6A and B, and their corresponding calculated proportions are included in Table 6. BEPI- and VAXI-vaccines were predicted to have 85% of the 3D residues exposed, which correlated with the result of the solvent accessibility analysis considering the secondary structure-based prediction. From the exposed residues, more than half were classified as polar residues (52% and 60% for BEPI- and VAXI-vaccines, respectively), with the VAXI-vaccine being predicted to have a higher polar profile. These results were concordant with the structure-dependent solubility analysis (Table 6). The BEPI-vaccine scored lower values (2.18 and 74.2%), while the VAXI-vaccine was predicted to be more soluble (2.45 and 81.1%). Structurally corrected solubility values above the threshold of 1 are considered highly soluble regions, while values below −1 are poorly soluble regions. Regarding the percentages, if the score is close to zero, the target protein is predicted as aggregation-prone, and when it is close to 130, as soluble. The structurally corrected solubility profile of BEPI- and VAXI-vaccines and the contribution of each single structural feature to the solubility prediction are detailed in Fig. S7A to D.

**Table 6.** Accessible surface area, polarity, and solubility of the final multi-epitope vaccine 3D models. Mol* 3D Viewer v5.0.0 was used to detect exposed/buried protein residues, and the CamSol v2.3 and SOLart v1.0 tools were used for solubility structure-based prediction. The best predicted scores for each characteristic between both vaccine constructs are boldfaced.

Vaccine characteristic	Feature specifications	BEPI-vaccine construct	VAXI-vaccine construct
Accessible surface area	Exposed residue proportion (percentage)	132/156 (85%)	105/124 (85%)
	Buried residue proportion (percentage)	24/156 (15%)	19/124 (15%)
Polarity	Polar residues/ER proportion (percentage)	69/132 (52%)	**62/105 (59%)**
	Non-polar residues/ER proportion (percentage)	63/132 (48%)	43/105 (41%)
Solubility	Structurally corrected solubility ^a^	2.18	**2.45**
	Solvent accessibility, backbone torsion angle, and inter-residue distance potentials	74.2%	**81.1%**

ER, exposed residues

^a^ Cutoff of the selection criteria of the immunoinformatics tool used for predictions: >1.

### Predicted immune response to BEPI- and VAXI-vaccines

We generated a computational hypothesis of the immune response triggered by the 2 multi-epitope vaccines with the C-ImmSim server simulation. It predicted that there would be a progressive increase of immunoglobulin M (IgM) and IgG concentrations during the immunization course, reaching the highest levels with the 3 vaccine doses (Fig. 5A and B). Compared with the primary immune response, the secondary immune response, which occurs as a result of subsequent exposure to the same antigen, was characterized by an almost 3-fold increase in IgM and IgG titers. However, the highest IgM titers predicted would be maintained between the second and third vaccination, especially in the case of the VAXI-vaccine (Fig. 5B), since IgM is the first antibody produced as a result of the first contact with an antigen. Regarding subtypes of IgG, the simulation showed also a peak of IgG1 and IgG2 together as well as only IgG1 after each immunization (Fig. 5A and B).

**Fig. 5.**
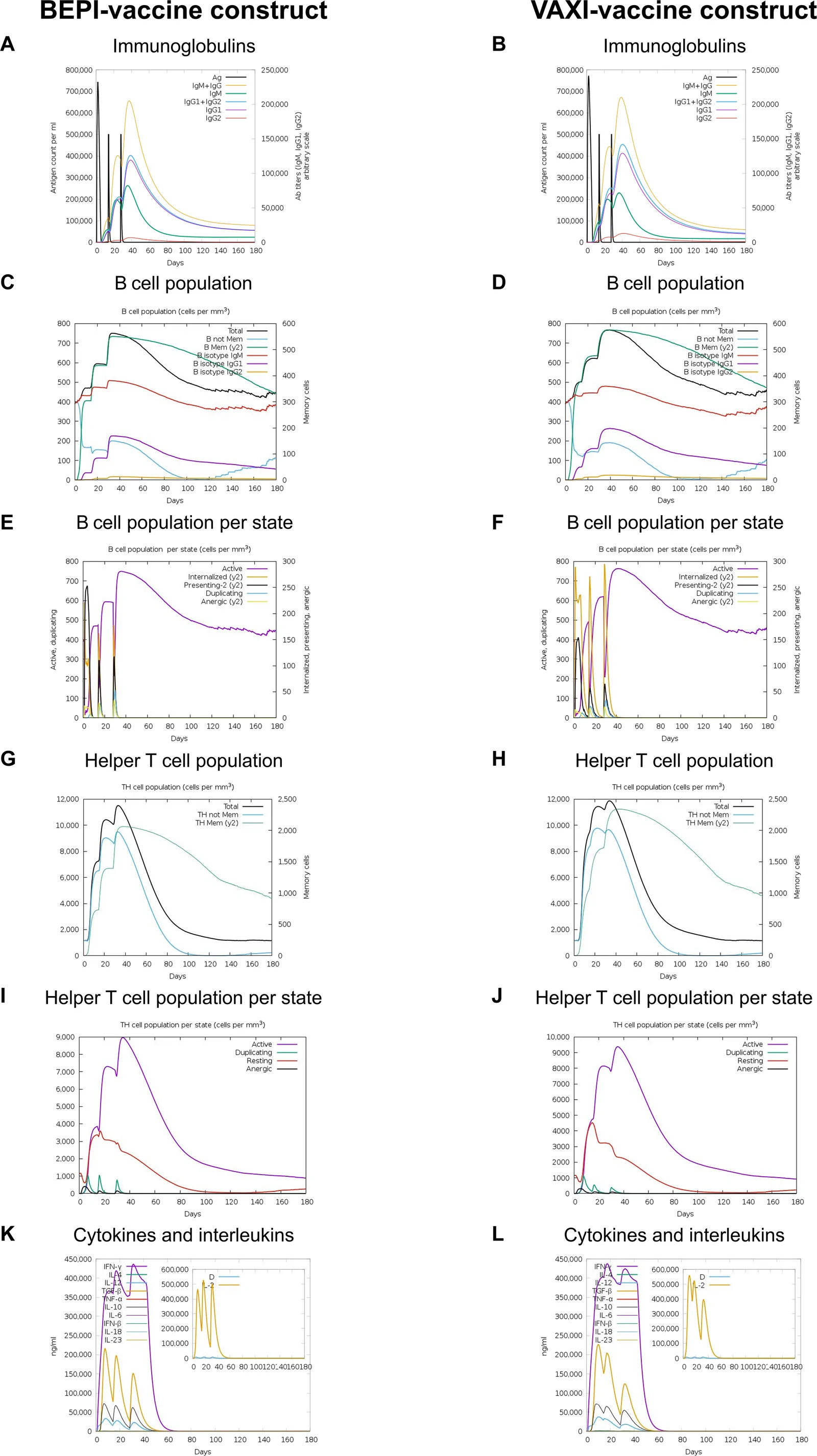
Simulated immune response upon BEPI and VAXI multi-epitope vaccine administration using C-ImmSim server. (A and B) Primary, secondary, and tertiary immune responses including immunoglobulin concentrations. (C and D) B cell populations. (E and F) B cell populations per state. (G and H) Helper T cell populations. (I and J) Helper T cell populations per state. (K and L) Concentrations of cytokines and interleukins including Simpson index “D” in the inset plots for BEPI- and VAXI-vaccine constructs.

These humoral response simulations would be in line with the higher number of memory B cells and active B cells predicted compared to other isotypes or functional states with the 3 vaccine doses, as shown in Fig. 5C to F. These 2 B cell populations were simulated to increase during the immunization course and would persist around half of the highest peak cell number recorded even 5 months after the last immunization, hypothesizing that a strong and long-lasting humoral response may be generated by both multi-epitope vaccines. Of note, levels of memory B cells generated by the VAXI-vaccine (Fig. 5D) would decrease more slowly over time after the last immunization compared to the population generated by the BEPI-vaccine (Fig. 5C).

Regarding cellular immunity, both multi-epitope vaccines would also promote helper T cell (Fig. 5G to J) and cytotoxic T cell responses (Fig. S8A to D), suggesting promising vaccine design approaches. The simulation predicted an approximately 1.5-fold increase of memory helper T cells with every vaccine dose, reaching higher levels with complete immunization with the VAXI-vaccine (around 11,500 cells per mm^3^) (Fig. 5H) than with the BEPI-vaccine (10,000 cells per mm^3^) (Fig. 5G). Memory helper T cells were also predicted to last several months in this simulated immunization contributing to longer protection. A similar pattern of active helper (Fig. 5I and J) and active cytotoxic (Fig. S8C and D) T cell populations was predicted to be developed by both vaccines, being activated in greater numbers by the VAXI-construct (Fig. 5J), around 1,000 cells per mm^3^ more compared to the BEPI-construct (Fig. 5I), with each vaccine dose.

Furthermore, the computational simulation showed that there would be an increased concentration of dendritic cells and macrophages. Most dendritic cells were predicted to rest during the immunization course (Fig. S8E and F), while within the macrophage population peaks of cells in an active state were predicted, with the antigen (vaccine construct) internalized or presenting it on the MHC-II after each immunization (Fig. S8G and H). These simulations suggest good antigen representation by these antigen-presenting cells.

Finally, the simulation data suggested that both vaccines would have the capacity to induce the production of cytokines and interleukins (Fig. 5K and L). The concentrations were predicted to be variable depending on the cytokine, with the highest levels reached by interferon-γ (IFN-γ), followed by transforming growth factor-β (TGF-β), and interleukin-10 (IL-10) and IL-12 (whose levels were approximately 2- to 8-fold change lower, respectively, compared to IFN-γ after each immunization). In the case of the simulated immunization with the VAXI-vaccine (Fig. 5L), between the first and second doses these levels would remain similar without a considerable decrease as occurred with the BEPI-vaccine after each immunization (Fig. 5K). The Simpson index D predicted a low level of danger when cytokine levels increased with every immunization, which supports that no complications may result during the immune response.

### Evaluation of vaccine population coverage

The ultimate goal of vaccine development is to achieve maximum population coverage. Our results showed that BEPI- and VAXI-vaccines were predicted to cover 99.99% and 99.98% of the world’s population with the combined MHC-I and MHC-II alleles, respectively (Fig. 6). Furthermore, considering separate MHC-I and MHC-II alleles, 89.85% and 99.92% for the BEPI-vaccine and 83.17% and 99.87% for the VAXI-vaccine were the population coverage percentages predicted worldwide, respectively (Table S2). Both vaccines showed a similar pattern across the 16 geographical regions analyzed, showing slight variation for some. The percentages of coverage with separate MHC-I and MHC-II alleles for each geographical area are detailed in Table S2. In conclusion, the included epitopes could also have the potential to induce T cell-mediated immunogenicity. Specifically, both multi-epitope vaccines were predicted to cover more than 80% of the world’s population considering any MHC allele calculation, and more than 75% regardless of the region and with class combined analysis.

**Fig. 6.**
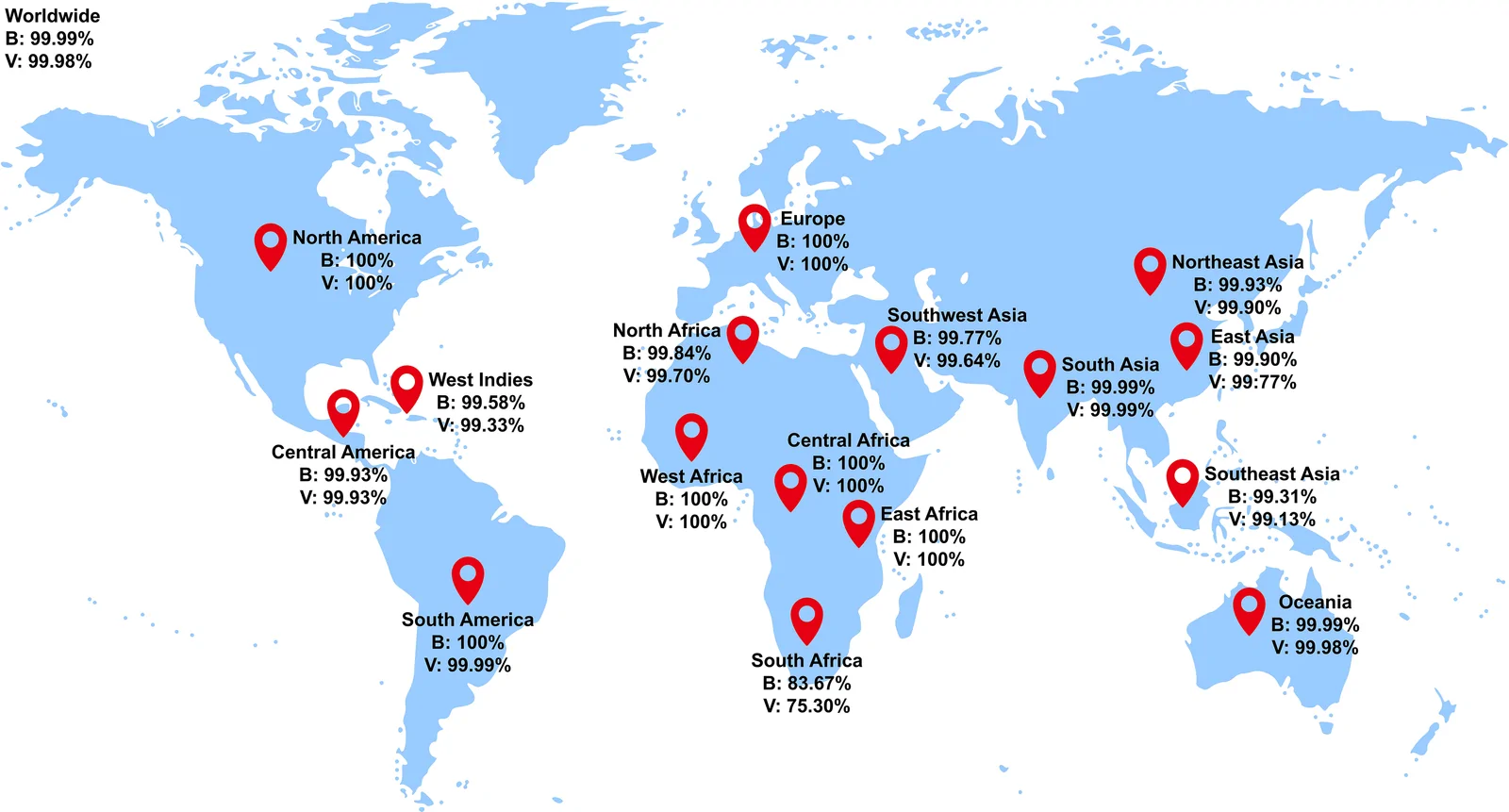
Population coverage of the BEPI and VAXI multi-epitope vaccines using IEDB v3.0.2 server. The reference sets of HLA class I and class II alleles with maximum population coverage and high or intermediate binding affinity for vaccine epitopes were used. The map only shows the population coverage results of the combined MHC-I and MHC-II alleles. B, BEPI-vaccine construct; V, VAXI-vaccine construct.

## Discussion

In the current challenging health scenario of multi-drug resistance surrounding *K. pneumoniae*, the development of a prophylactic vaccine against this pathogen may represent a promising strategy to prevent infections. However, conventional vaccine development involves time-consuming, costly, and laborious *in vivo* and *in vitro* methodologies [21,42,45,46,53], which delays potential application of new vaccines in humans. In addition to this, the traditional whole-organism-based vaccines have the potential to induce allergic responses and may involve inadvertent antigen exposure resulting in ineffective recognition [29,45].

Advancements in the fields of bioinformatics and immunoinformatics, supported by public and updated genomic and proteomic databases comprising a wide range of microorganisms and strains, are enabling faster vaccine development by detecting many potential vaccine candidates and screening for ideal epitopes. Targeting only the antigenic parts of the protein is expected to lead to more intensive immune responses and lower reactivity [40,41,47,55]. The success in computational design, considering specificity, simplicity, safety, and accuracy, and a more convenient production process of the multi-epitope vaccines have attracted the scientific community [40,53,84,140], as evidenced by the large number of studies using this strategy against different infections and diseases.

In this study, we have used *in silico* approaches to design and assess immunogenicity of 2 multi-epitope vaccines following different criteria in the selection of B cell epitopes to identify antigenic regions most likely to induce antibody-mediated recognition of *K. pneumoniae* target proteins. Our decision to focus primarily on B cell epitopes is grounded in the pathobiology of *K. pneumoniae* as an extracellular pathogen, where humoral immunity plays the predominant protective role and is considered a key component of protective immunity. As noted by Paczosa and Mecsas [3], immunity against *K. pneumoniae* is well-characterized as being heavily dependent on antibody-mediated responses, which are essential for opsonization, Fc receptor-dependent phagocytosis, complement activation, and neutralization of surface virulence factors. Previous experimental studies with *K. pneumoniae* proteins including MrkA [58,143] and FepA [31] have demonstrated that antibody responses alone can confer significant protection. Although the prediction of B cell epitopes is much more complex than the prediction of T cell epitopes, due to variability in epitope length from 3 to 85 amino acids [90], we focused on B cells as key elements that trigger the protective humoral immune response described above [3]. As an extracellular pathogen, immunity against *K. pneumoniae* also requires helper T cells that contribute to sustained immune responses, preventing further dissemination within the host or the development of associated pathologies [3]. For this purpose, we included the PADRE peptide within the vaccine constructs, which combines potent helper T cell activity, a broad range of binding to mouse and human MHC-II alleles, and safety and good tolerability in human clinical studies [35,50,144]. In addition, beyond providing broad CD4^+^ T cell help, PADRE peptide-mediated activation can enhance B-cell stimulation and cytokine production and support the development of robust humoral and cellular immune responses. However, we acknowledge that the PADRE peptide cannot fully substitute for pathogen-derived CD4^+^ T cell epitopes capable of eliciting antigen-specific cellular immunity, effective antigen processing and presentation, germinal center formation, affinity maturation, and long-term immunological memory. On the other hand, CD8^+^ T cell epitopes were not included because our design strategy was primarily focused on optimizing humoral immunity against extracellular virulence factors. Besides, prediction of CD8^+^ T cell epitopes was not considered for our vaccine formulations since cytotoxic T cells can induce adverse inflammation-related pathologies in certain conditions, as demonstrated by the increase of highly inflammatory cytotoxic CD57^+^CD8^+^ T cell subsets, which are associated with short-term cardiovascular mortality in patients with acute myocardial infarction [145], after immunization with a multi-bacterium vaccine including *K. pneumoniae* [146]. Given these limitations of our study, future vaccine optimization should therefore integrate predictions of conformational B cell epitopes derived from candidate proteins and experimentally validated or computationally predicted T cell epitopes to enhance immunogenicity, population coverage, and long-term protective efficacy.

In our approach, we have also incorporated different *K. pneumoniae* virulence factors into a single epitope-based vaccine with the aim of improving protection coverage given the high variability among strains. In line with this, our results indicated that the selected antigens are fully conserved in 76% to 96% of analyzed strains when analyzing them independently, representing over 250 isolates from different samples and locations. The computational design of our vaccines combines epitopes belonging to empirical protective proteins and potential antigenic proteins identified using immunoproteomic technologies, which could enhance the probability of protection of the designed vaccines and their potential to provide broad-spectrum protection, respectively [21].

First, we assessed immunogenicity of the original candidate proteins, and we observed that there was a score variability ranging from 0.60 to 0.79, with no correlation between the score and the size nor the functional category of the protein, indicating the broad antigenicity potential of *K. pneumoniae* surface proteins. Since all proteins were predicted to have antigenic potential, we can assume that the use of different virulence factors from a target microorganism may represent a more comprehensive approach to vaccine construction.

Among the proteins with the highest antigenicity score was MrkA, the major subunit of type 3 fimbriae involved in biofilm formation, allowing *K. pneumoniae* to persist on medical devices and hospital surfaces and to attach lung epithelial cells. High scores were also observed for LamB and PhoE, 2 porins whose loss is associated with resistance to β-lactam antibiotics, suggesting their potential as novel antigens. FepA, one of the first iron uptake system proteins identified in *E. coli*, with an essential role in establishing infection in the host, also obtained a high antigenicity score. In accordance with this, the highest number of predicted B cell epitopes was obtained for the most antigenic proteins mentioned. It is worth noting that MrkA has been predicted to have a considerable number of B cell epitopes despite its shorter length, suggesting great potential for being used as a protective antigen.

From the list of proteins of our study, some have already been tested *in vivo* as vaccine candidates. Active or passive immunization protocols have been carried out, using the *K. pneumoniae* protein or its homolog from *E. coli* of MrkA [58,143,147], OmpW [148], FepA [31,149,150], and PhoE [151], with promising data in terms of antibody response, clearance of bacterial load, or protection. Recombinant OmpW belonging to *A. baumannii*, another critical priority pathogen considered by the WHO, has also been successfully tested in mouse model [67]. Therefore, there could be a correlation between our predicted *in silico* results regarding antigenicity and the *in vivo* data available for these proteins. A recent and comprehensive study by Shahbazi *et al*. [64] shows that immunization with *K. pneumoniae* OmpA protein elicits significant production of specific total IgG antibodies. However, the lack of demonstrated protective efficacy may explain the lowest predicted score as a probable protective antigen. Furthermore, the most antigenic epitope predicted in OmpA is an internal loop localized in the periplasm, revealing the importance of structure-based analysis for determining exposed antigenic epitopes. Our assumption may be supported by another study showing significant protection and higher antibody levels using an OmpA-OmpK36 fusion protein vaccine [61], which could suggest that the major role in the development of effective immunity may partly derive from the contribution of OmpK36 protein. For LamB, FhuA, and Fiu proteins, there are no experimental immunization studies to date, which merits further investigation. In addition, how the expression of CPS affects the ability of vaccine-induced antibodies to bind to their corresponding protein antigen will require further study, as it is currently unknown how capsule affects accessibility of the selected epitopes.

Since B cell epitope prediction has been the target for multiple immunoinformatics improvements to elucidate the complexity in the interaction between antigenic epitope and antibody paratope [43,144,152], we revalidated our B cell epitope selection strategies. Linear B cell epitopes predicted following the BEPI- and VAXI-strategies were supported by additional analyses that considered structural and conformational features. Both multi-epitope sequences were composed of 3D epitopes, being exposed and antigenic.

The protective antigenic profile of our 2 developed multi-epitope vaccines was predicted to be enhanced in comparison with the original proteins, as was consistently confirmed in the case of the BEPI-vaccine. Although the antigenic potential of the VAXI-vaccine was greater, it was only supported by one prediction tool. This reveals the importance of using 2 or more bioinformatics approaches to obtain reliable predictions. A limitation of the bioinformatics approach used in this study is the absence of molecular docking of the vaccine constructs with Toll-like receptors (e.g., TLR2 and TLR4) or molecular dynamics simulations. Although these analyses are not mandatory in computational vaccine design studies, they are frequently used to provide preliminary insights into the interaction of vaccine–receptor complexes, their stability, and putative immune activation mechanisms.

If we compare the 2 multi-epitope vaccines with respect to their physicochemical characteristics, both vaccine proteins were predicted to be efficiently expressed and structurally soluble (without a presumably aggregation-prone profile) and stable. Specifically, the BEPI-vaccine could have the benefit of better thermostability facilitating its purification at room temperature, while the VAXI-vaccine would be more hydrophilic through sequence- and structure-based solubility analyses. A challenging issue for protein-based therapeutics lies in their inadequate developability, which includes physical and/or chemical factors (stability, aggregation tendency, solubility, concentration, or viscosity), diverse surface properties, and complex formulations [84]. The small size, high degree of solubility, hydrophilic nature, and considerable stability of our 2 vaccine proteins make them ideal for enhanced purification during the downstream manufacturing process, being key factors in vaccine post-production studies.

Using the most antigenic epitopes of a target protein to construct a vaccine (like our VAXI-strategy) may have adverse effects, potentially affecting in terms of toxicity, and theoretically increase the risk of undesired biological effects. Nevertheless, none of the vaccines created *in silico* in this study were predicted to provoke allergenic, toxic, or autoimmune reactions in the body, supporting their potential future use in humans.

Regarding the simulated immune response, our hypothesis is that the VAXI-vaccine stands out over the BEPI-vaccine, as it was predicted to produce higher levels of IgM indicating an earlier recognition of the antigen. In this line, it is assumed that the VAXI-vaccine would be better recognized by B cells than the BEPI-vaccine, as evidenced by the higher peaks of B cells with the internalized antigen predicted after each immunization. Our assumption may be supported by the increased hydrophilicity of the VAXI-vaccine protein and the lower proportion of predicted buried residues in its secondary and tertiary structures. In fact, the VAXI-vaccine construct would have a higher proportion of polar amino acids exposed in a conformational structure than the BEPI-vaccine. It has been demonstrated that mutations from hydrophilic to hydrophobic amino acids can lead to reduced antibody binding ability and immunogenicity [153]. Furthermore, the topological accessibility of B cell epitopes to antibodies during bacterial infections plays a pivotal role in developing protection [33]. In this regard, both vaccine proteins were predicted to have a large percentage of accessible surface area. Therefore, considering all these physicochemical and structural aspects that affect immunogenicity, we hypothesize that the VAXI-vaccine could induce a better memory B and T cell response, leading to greater protective efficacy against future infections. Interestingly, the cytokine profile predicted for both vaccines might also suggest the activation of mucosal immunity, particularly due to the higher levels of TGF-β and IL-10 compared to other cytokines observed. *K. pneumoniae* is a common colonizing pathogen of the gastrointestinal, respiratory, and genitourinary mucosae in humans [28]. The potential induction of mucosal IgA responses, alongside systemic IgG responses, could beneficially contribute to preventing colonization.

The 3D structures of vaccines can represent a key factor in their biological significance. It is acknowledged that, in AlphaFold, disordered regions are often represented by large loops that interfere with the overall geometry of the protein, resulting in unrealistic structures [126,142,154]. In this regard, the Robetta models might be more reliable, as the *de novo* structure prediction method enabled the generation of improved models by resolving all the loops without major perturbation of the core α-helix organization.

Given that population coverage aims to estimate the proportion of individuals within a population capable of presenting selected epitopes via HLA molecules, the high percentage of population coverage around the world revealed not only the potential of both vaccines to protect people but also the potential of the selected peptide regions to bind to HLA molecules and, consequently, to receive T cell help following antigen processing. Adequate HLA presentation could be assumed, as we selected only those HLA alleles that had previously shown high or intermediate binding affinity for the vaccine peptide sequences. Other studies that also used different antigen proteins to construct a *K. pneumoniae* multi-epitope vaccine showed similar or lower percentages of coverage [29,55], which may support the effectiveness of our B cell epitope-based design strategy combined with the inclusion of the PADRE peptide. In this sense, the highly conserved *K. pneumoniae* epitopes used in our vaccine constructs could potentially help to achieve global protection. However, because the vaccine design did not explicitly incorporated antigen-derived MHC-I- and MHC-II-restricted T cell epitopes, the reported population coverage values may overestimate the biological significance of the multi-epitope vaccine constructs with respect to cellular immunity.

Our study reveals distinct advantages for each construct depending on the evaluation criterion. Regarding predicted immunogenicity, the VAXI-vaccine demonstrated superior antigenic potential (VaxiJen v2.0 score: 1.37 *vs*. 0.91 for the BEPI-vaccine), although this was supported by only one of the 2 prediction tools, with ANTIGENpro favoring the BEPI-vaccine (0.85 *vs*. 0.66). If we pay attention to the 3D antigenic epitopes, the VAXI-vaccine also contained slightly higher proportion of exposed antigenic residues according to SEPPA 3.0 and SEMA v2.0 predictions (113/124 and 117/124) than the BEPI-vaccine (141/156 and 143/156), respectively, which strengthens the VaxiJen v2.0 result. The *in silico* immune simulation results favored the VAXI-vaccine, which was predicted to induce higher memory B cell levels (approximately 600 cells/mm^3^), more sustained IgM titers between immunizations, and elevated helper T cell responses (11,500 *vs*. 10,000 cells/mm^3^ for memory T helper cells). Importantly, the VAXI-vaccine’s higher hydrophilicity (GRAVY: −0.92 *vs*. −0.70) and greater proportion of surface-exposed residues correlate with enhanced B cell recognition, as hydrophilic residues improve antibody binding capacity [153]. In terms of stability, both constructs were predicted to perform favorably with IIs around 27 (well below the threshold of 40), although the BEPI-vaccine showed marginally better thermostability (AI: 47.69 *vs*. 45.00), potentially facilitating room-temperature purification.

As conclusions from this comparative study of *in silico* approaches to construct vaccines, both strategies follow appropriate criteria, and their results are supported by one or 2 bioinformatics tools. Specifically, we used a comprehensive computational workflow incorporating multiple independent tools for epitope prediction, topology analysis, conservation assessment, structural modeling, physicochemical characterization, allergenicity prediction, toxicity prediction, immune simulation, and population coverage analysis to increase confidence in the computational predictions of the designed multi-antigen vaccine candidates. The BEPI-strategy ensures the correct identification of B cell epitopes for vaccine construction supported by containing the highest probability residue, while the VAXI-strategy selects epitopes considering their potential as protective antigens, which is a key factor for human application. Previous studies comparing different vaccine design strategies [29,78,79] relied on positive *in silico* selection criteria to determine the most optimal design (including physicochemical properties, solubility, antigenicity, or Ramachandran analysis). However, unlike those studies, the BEPI- and VAXI-strategies were compared across all bioinformatics and immunoinformatics analyses conducted in this work, thereby reinforcing the objective of identifying the most promising construct within the fully applied computational framework. According to this, the VAXI-vaccine emerges as the most promising candidate to continue further studies, since it meets all the favorable conditions for production and is predicted to develop better immunity. Nevertheless, it is acknowledged that *in silico* prediction tools offer only valuable guidance for candidate prioritization. Consequently, the predicted modest advantages of the VAXI-vaccine construct (in terms of antigenicity, solubility, and simulated immune responses) should be considered as hypothesis-generating rather than indicative of biological superiority in vaccine performance. Beyond these *in silico* predictions, subsequent structural confirmation including molecular docking will be required for both vaccine constructs.

Given the inherent limitations of immune simulation platforms, further studies are needed to validate the *in silico* results of our multi-epitope vaccines. The potential next steps may include, for example, recombinant expression of the vaccine proteins, immunogenicity studies in suitable animal models, enzyme-linked immunosorbent assay (ELISA)-based antibody characterization, bacterial challenge experiments, complement-mediated bactericidal assays, and opsonophagocytic killing assays. Validation of *in silico*-designed vaccines by subsequent *in vivo* studies has been successfully achieved by previous studies [32,35,38,39,46,48,50], but it is required as some predictions may fail in physiological conditions [26,33]. However, Liao *et al*. [27] recently designed a multi-epitope vaccine, which includes epitopes from different antigens, and proved this strategy to be successful *in vivo* as it elicited robust protective efficacy against *K. pneumoniae* lung infection. It is noteworthy that the epitopes extracted from OmpA, OmpW, and FepA proteins included in our work (except the periplasmic epitope from OmpA) have also been identified and used in this *in vivo* study. This provides strong external validation and highlightsthe reliability of the approaches followed for *in silico* development of the BEPI- and VAXI-vaccines, thereby supporting theirfurther investigation and their promising applicability. Our work aligns with and extends several recent studies. Wang *et al.* [21] employed a pangenome-immunoinformatics approach targeting 4 specific capsular serotypes (K1, K2, K47, and K64), while our strategy focuses on conserved surface virulence factors to potentially achieve broader serotype coverage. Cuscino *et al.* [26] utilized reverse vaccinology against carbapenemase-producing strains, and Hakimian *et al.* [29] compared adjuvant positioning, whereas our comparative BEPI- *vs*. VAXI-strategy specifically addresses epitope selection criteria, a methodological dimension not previously explored systematically. Our inclusion of the LamB and PhoE porins is particularly relevant given the findings of Tajuelo *et al.* [20], whose deep intraclonal analysis emphasized the importance of targeting structures associated with antimicrobial resistance mechanisms. The study by Prakoso *et al.* [155] highlighted the role of OMPs as candidate target proteins for developing a multi-epitope vaccine against *K. pneumoniae*. Likewise, Li *et al.* [79] and Ballim *et al.* [81] have adopted a complex approach targeting up to 6 outer membrane proteins of *K. pneumoniae* (including OmpA and OmpW) and encompassing B and T cell epitopes. Interestingly, through physicochemical properties and antigenicity analysis, Li *et al.* ruled out PhoE and LamB porins as candidate proteins. Their potential may have been underestimated, given that our VAXI-vaccine construct achieved higher antigenicity and solubility scores than the best vaccine candidate they proposed (using the same prediction tools, VaxiJen v2.0 and SOLpro, respectively). Siderophore receptors constituted another functional protein category of our study, representing also the approach proposed by Elfadil *et al*. [156] to specifically combat hypervirulent NUBRI-K strain. The solubility of the resulting multi-epitope vaccine construct, as predicted by SOLpro, was lower than the BEPI- and VAXI-vaccines, which could be attributed to the rigidity of the incorporated adjuvant (cholera toxin B subunit) [79,156]. Likewise, *K. pneumoniae* type 1 fimbriae proteins were recently the subject of a study conducted by Chegene Lorestani *et al.* [78] for immunoinformatics purposes. Afshan *et al*. [157] pursued an innovative strategy that incorporated multiple antigens from *K. pneumoniae* and *A. baumannii* into a single vaccine construct with the aim of combating co-infection. To counteract the high polymorphism of HLA class II alleles and enhance immunogenicity, several of these recent studies also incorporated the PADRE peptide into the vaccine construct [78,155,157], thereby supporting our approach. Collectively, our findings contribute to the growing consensus that multi-epitope vaccines targeting conserved virulence factors represent a promising strategy for addressing the therapeutic challenges posed by multidrug-resistant and hypervirulent *K. pneumoniae* strains, as reviewed by Douradinha [18] and Dangor *et al.* [13].

## Conclusions

*K. pneumoniae* is a difficult-to-treat pathogen due to its highly antimicrobial resistance and hypervirulent phenotypes acquired in recent decades. The development and improvement of bioinformatics tools have enabled the rapid identification and prioritization of potential antigens of this species for *in silico* vaccine design, and selecting the most promising immunogens for subsequent experimental validation, which reduces greatly the costs and time associated with their validation. In this study, 2 immunoinformatics approaches to construct multi-epitope vaccines based on B cell epitope prediction have been compared. Furthermore, 3 virulence factors found in *K. pneumoniae*, such as a type 3 fimbrial subunit, porins, and iron uptake systems, have been included in the design process to improve the vaccine coverage and reduce the incidence of infections in hospital and community settings. Both strategies have been based on reliable criteria and have resulted in multi-epitope vaccines with appropriate physicochemical characteristics and promising immunogenic action, postulating them as potential vaccine candidates. Future wet laboratory validation and *in vivo* studies are the next steps required to verify the current findings with the aim to evaluate their preventive and/or therapeutic application in humans.

## Data Availability

The datasets generated during and/or analyzed during the current study are available from the corresponding author on reasonable request.

## Supplementary Materials

Supplementary 1 Textfiles S1 and S2 Figs. S1 to S8 Tables S1 to S3
