# Supplementary material for: In Silico Design and Immunogenicity Studies of Two Multi-Epitope Vaccines against Klebsiella pneumoniae
Source: Comput Struct Biotechnol J. 2026 Oct 28;35(1):0232. doi: 10.34133/csbj.0232 (PMC13714322; doi:10.34133/csbj.0232)
Supplement: Supplementary file 1 [file csbj.0232.f1.docx]

**Supplementary material**

*In silico* design and immunogenicity studies of two multi-epitope vaccines against *Klebsiella pneumoniae*

Marta Vicente-Pazos ^a,b,c^, Mireia López-Siles ^c,#,^*, and Michael J. McConnell ^a,d,e,#^

a. Intrahospital Infections Laboratory, National Centre for Microbiology, Instituto de Salud Carlos III (ISCIII), Madrid, Spain.
b. Doctoral Program in Biomedical Sciences and Public Health, IMIENS, Universidad Nacional de Educación a Distancia (UNED), Madrid, Spain ([mvicente488@alumno.uned.es](mailto:mvicente488@alumno.uned.es)).
c. Microbiology of Intestinal Diseases, Biology Department, Universitat de Girona, Girona, Spain ([mireia.lopezs@udg.edu](mailto:mireia.lopezs@udg.edu)).
d. Department of Biological Sciences, University of Notre Dame, Notre Dame, Indiana, USA ([mmcconn9@nd.edu](mailto:mmcconn9@nd.edu)).
e. Eck Institute for Global Health, Notre Dame, Indiana, USA.

\# These authors contributed equally to this work and share the last authorship.

***Correspondence to:**

Mireia López-Siles

[mireia.lopezs@udg.edu](mailto:mireia.lopezs@udg.edu)

**Supplementary Tables**

**Supplementary Table S1. List of predicted linear B cell epitopes for each selected candidate protein using BepiPred-3.0 server.** No, number; and BCE, B cell epitope.

|  | **Local linear BCE prediction** | | | | |  |
| --- | --- | --- | --- | --- | --- | --- |
| **Protein** | **No** | **Peptide** | **Start** | **End** | **Length** |  |
| **MrkA** ^a,c^ | 1 | DTTVGG | 24 | 29 | 6 |  |
|  | 2 | GQGSDA | 49 | 54 | 6 |  |
|  | 3 | LTEVKAAAADTYLKP | 63 | 77 | 15 |  |
|  | 4 | CQAADGTKQDDVS | 87 | 99 | 13 |  |
|  | 5 | GNLLAGATSKQQGYLANTEASGA | 108 | 130 | 23 |  |
|  | 6 | NATALTNKIIPGDSTQPKAKGDASAVADG | 141 | 169 | 29 |  |
|  | 7 | TSAPTTVTT | 180 | 188 | 9 |  |
| **OmpA** ^a,c^ | 1 | DTGFYGNGFQNNNGPTRN | 41 | 58 | 18 |  |
|  | 2 | KGNYASTGVSRSEHD | 133 | 147 | 15 |  |
|  | 3 | DAAPVVAPAPAPAPEVAT | 205 | 222 | 18 |  |
|  | 4 | NTCDNVKARAALI | 319 | 331 | 13 |  |
|  | 5 | EVVTQ | 349 | 353 | 5 |  |
| **OmpW** ^a,c^ | 1 | TEGSDNVLGSLGSFNVS | 39 | 55 | 17 |  |
|  | 2 | KVGTGPTGT | 85 | 93 | 9 |  |
|  | 3 | NEDFNDTGKAAGLSDLSLK | 132 | 150 | 19 |  |
|  | 4 | KFKAGGVDQKVST | 185 | 197 | 13 |  |
| **LamB** ^a,c^ | 1 | VNQEDDWESTS | 93 | 103 | 11 |  |
|  | 2 | SGGSYTFSSDDTKKYAA | 174 | 190 | 17 |  |
|  | 3 | PQDDYRLEDGA | 223 | 233 | 11 |  |
|  | 4 | NSGHSQGTSI | 268 | 277 | 10 |  |
|  | 5 | LDSKN | 311 | 315 | 5 |  |
|  | 6 | SQRTSE | 347 | 352 | 6 |  |
|  | 7 | NTSGLQTKDSSGS | 394 | 406 | 13 |  |
| **PhoE** ^a,c^ | 1 | SDYDSKDGD | 45 | 53 | 9 |  |
|  | 2 | GNKTESDSSQK | 83 | 93 | 11 |  |
|  | 3 | DMFPEFGGDSSAQTDNFMT | 126 | 144 | 19 |  |
|  | 4 | NEGREAKKQN | 175 | 184 | 10 |  |
|  | 5 | RTNDQNLLARGQGSK | 212 | 226 | 15 |  |
|  | 6 | MTPISGGFA | 253 | 261 | 9 |  |
|  | 7 | GKDIEGVGSEDL | 290 | 301 | 12 |  |
|  | 8 | LKSDNKLGIND | 328 | 338 | 11 |  |
| **FepA** ^b,d^ | 1 | NMTEQSTPDESAAT | 27 | 40 | 14 |  |
|  | 2 | NAGHEAERTGSYAGSY | 240 | 255 | 16 |  |
|  | 3 | AGDTQNTNTSTLVKSM | 295 | 310 | 16 |  |
|  | 4 | SRMDEGLAGGTEGIFSSS | 349 | 366 | 18 |  |
|  | 5 | TQALSYGTIDGVSATGRSPYS | 412 | 432 | 21 |  |
|  | 6 | NGQGCYA | 510 | 516 | 7 |  |
|  | 7 | TASNG | 572 | 576 | 5 |  |
|  | 8 | KRFNYKGEAVSGS | 662 | 674 | 13 |  |
|  | 9 | TGNATTGAYL | 720 | 729 | 10 |  |
| **Fiu** ^b,d^ | 1 | ANNITAPDI | 346 | 354 | 9 |  |
|  | 2 | GLDRNGANA | 432 | 440 | 9 |  |
|  | 3 | SATACGGSGRGAIACPPGQSTGSPVTTVDTAKS | 478 | 510 | 33 |  |
|  | 4 | GAVGTP | 694 | 699 | 6 |  |

Cutoffs of the predicted BCE for each protein: ^a^ >0.1512 and ^b^ >0.0802 (epitope screening); and ^c^ ~0.2 and ^d^ ~0.12 (epitope selection).

**Supplementary Tables S2. Population coverage calculation result for the BEPI and VAXI multi-epitope vaccines using the IEDB v3.0.2 population coverage analysis tool.** Population coverage rates were estimated independently for each vaccine worldwide and in 16 geographical areas using the reference sets of HLA class I and class II alleles with maximum population coverage and high or intermediate binding affinity for vaccine epitopes.

| **BEPI-vaccine construct** | **Class I** | | | **Class II** | | | **Class combined** | | |  |
| --- | --- | --- | --- | --- | --- | --- | --- | --- | --- | --- |
| **Geographical area** | Coverage ^a^ | Average hit ^b^ | pc90 ^c^ | Coverage ^a^ | Average hit ^b^ | pc90 ^c^ | Coverage ^a^ | Average hit ^b^ | pc90 ^c^ | |
| **Worldwide** | 89.87% | 11.77 | 7.9 | 99.92% | 35.89 | 25.34 | 99.99% | 47.66 | 34.65 | |
| **Oceania** | 65.92% | 6.54 | 2.35 | 99.97% | 35.38 | 25.57 | 99.99% | 41.92 | 30.52 | |
| **South America** | 70.68% | 7.47 | 2.73 | 99.98% | 39.09 | 28.4 | 100.0% | 46.55 | 34.19 | |
| **Central America** | 2.78% | 0.22 | 0.82 | 99.93% | 26.1 | 18.39 | 99.93% | 26.32 | 18.49 | |
| **North America** | 90.24% | 11.98 | 8.05 | 100.0% | 41.61 | 32.56 | 100.0% | 53.59 | 41.71 | |
| **West Indies** | 84.52% | 10.5 | 5.17 | 97.26% | 20.14 | 11.79 | 99.58% | 30.64 | 19.73 | |
| **South Africa** | 75.95% | 8.43 | 3.33 | 32.1% | 2.66 | 1.18 | 83.67% | 11.09 | 4.9 | |
| **North Africa** | 78.83% | 9.0 | 3.78 | 99.25% | 26.24 | 17.21 | 99.84% | 35.24 | 24.32 | |
| **Central Africa** | 64.19% | 6.86 | 2.23 | 99.99% | 40.53 | 30.3 | 100.0% | 47.4 | 34.9 | |
| **West Africa** | 76.01% | 8.88 | 3.33 | 99.99% | 40.89 | 31.31 | 100.0% | 49.78 | 37.34 | |
| **East Africa** | 74.07% | 8.03 | 3.09 | 99.99% | 42.0 | 32.0 | 100.0% | 50.03 | 37.39 | |
| **Europe** | 95.43% | 14.07 | 9.28 | 100.0% | 37.94 | 28.03 | 100.0% | 52.01 | 40.43 | |
| **Southwest Asia** | 78.02% | 8.69 | 3.64 | 98.93% | 22.53 | 14.48 | 99.77% | 31.22 | 20.52 | |
| **Southeast Asia** | 71.06% | 7.26 | 2.76 | 97.6% | 21.27 | 12.35 | 99.31% | 28.53 | 17.96 | |
| **South Asia** | 82.62% | 9.6 | 4.6 | 99.96% | 33.26 | 24.37 | 99.99% | 42.86 | 31.89 | |
| **Northeast Asia** | 81.64% | 9.35 | 4.36 | 99.64% | 30.26 | 19.78 | 99.93% | 39.61 | 27.18 | |
| **East Asia** | 81.69% | 10.26 | 4.37 | 99.45% | 27.42 | 17.87 | 99.9% | 37.69 | 25.59 | |

| **VAXI-vaccine construct** | **Class I** | | | **Class II** | | | **Class combined** | | |  |
| --- | --- | --- | --- | --- | --- | --- | --- | --- | --- | --- |
| **Geographical area** | Coverage ^a^ | Average hit ^b^ | pc90 ^c^ | Coverage ^a^ | Average hit ^b^ | pc90 ^c^ | Coverage ^a^ | Average hit ^b^ | pc90 ^c^ | |
| **Worldwide** | 83.17% | 10.14 | 4.75 | 99.87% | 34.1 | 24.09 | 99.98% | 44.24 | 31.94 | |
| **Oceania** | 60.75% | 6.02 | 2.04 | 99.96% | 34.46 | 25.02 | 99.98% | 40.49 | 28.88 | |
| **South America** | 67.06% | 6.91 | 2.43 | 99.98% | 38.25 | 27.69 | 99.99% | 45.16 | 33.23 | |
| **Central America** | 4.14% | 0.33 | 0.83 | 99.93% | 25.74 | 18.19 | 99.93% | 26.08 | 18.33 | |
| **North America** | 85.07% | 10.5 | 5.36 | 100.0% | 39.56 | 30.0 | 100.0% | 50.06 | 38.21 | |
| **West Indies** | 80.83% | 9.92 | 4.17 | 96.52% | 19.04 | 10.9 | 99.33% | 28.96 | 18.22 | |
| **South Africa** | 73.25% | 8.44 | 2.99 | 7.65% | 0.62 | 0.87 | 75.3% | 9.06 | 3.24 | |
| **North Africa** | 75.08% | 8.51 | 3.21 | 98.81% | 24.33 | 16.02 | 99.7% | 32.84 | 21.37 | |
| **Central Africa** | 62.08% | 6.5 | 2.11 | 99.99% | 38.93 | 28.65 | 100.0% | 45.43 | 33.53 | |
| **West Africa** | 71.41% | 8.03 | 2.8 | 99.99% | 39.95 | 30.14 | 100.0% | 47.97 | 35.63 | |
| **East Africa** | 72.82% | 8.23 | 2.94 | 99.99% | 40.11 | 29.66 | 100.0% | 48.34 | 35.57 | |
| **Europe** | 88.71% | 11.84 | 7.08 | 100.0% | 35.89 | 26.14 | 100.0% | 47.73 | 35.31 | |
| **Southwest Asia** | 70.31% | 7.64 | 2.69 | 98.77% | 21.69 | 13.64 | 99.64% | 29.33 | 18.82 | |
| **Southeast Asia** | 69.73% | 7.28 | 2.64 | 97.13% | 20.3 | 11.58 | 99.13% | 27.59 | 17.27 | |
| **South Asia** | 75.92% | 8.63 | 3.32 | 99.94% | 31.68 | 22.55 | 99.99% | 40.31 | 28.49 | |
| **Northeast Asia** | 78.03% | 8.39 | 3.64 | 99.56% | 29.27 | 19.01 | 99.9% | 37.66 | 25.8 | |
| **East Asia** | 68.11% | 7.13 | 2.51 | 99.29% | 26.47 | 17.16 | 99.77% | 33.61 | 22.15 | |

^a^ projected population coverage; ^b^ average number of epitope hits / HLA combinations recognized by the population; ^c^ minimum number of epitope hits / HLA combinations recognized by 90% of the population.

**Supplementary Table S3. Bioinformatic and immunoinformatic servers used in the study.** The table includes the software version, parameter settings, and required input file for each tool employed. The input file is specified based on the analysis performed for the candidate protein, epitope, or multi-epitope vaccine construct. -, no necessary parameter settings.

| **Tool** | **Parameter settings** | **Input file** |
| --- | --- | --- |
| **BepiPred-3.0** | Top epitope percentage cutoff: Higher confidence (top 20%)  Threshold: 0.1512 / 0.0802 | Protein sequence (FASTA format) |
| **iBCE-EL** | - | Epitope sequence (FASTA format) |
| **SEPPA 3.0** | Chain: A  Subcellular localization of antigen: Secreted  Species of the immune host: Homo  Threshold: 0.089 | Refined 3D structure of the multi-epitope vaccine (PDB file) |
| **SEMA v2.0** | SEMMA-1D | Multi-epitope vaccine sequence (Plain format) |
| **DeepTMHMM**  **version 1.0.42** | - | Protein sequence (FASTA format) |
| **BOCTOPUS 2** | - | Protein sequence (FASTA format) |
| **Epitope Conservancy Analysis** | Analysis type: Epitope linear sequence conservancy  Sequence identity threshold: ≥90% / ≥100%  Remove duplicated protein sequences: Deactivated | Protein sequence, epitope sequence (FASTA format) |
| **JCat** | Sequence type: Protein sequence  Organism: *E. coli* strain K12 ^a^ / *H. sapiens*  Avoid rho-independent transcription terminators: Activated  Avoid prokaryotic ribosome binding sites: Activated  Avoid cleavage sites of restriction enzymes: Deactivated | Multi-epitope vaccine sequence (FASTA format) |
| **Emboss backtranseq** | Codon table: *H. sapiens* | Multi-epitope vaccine sequence (FASTA format) |
| **Expasy ProtParam** | - | Multi-epitope vaccine sequence (Plain format) |
| **SOLpro** | - | Multi-epitope vaccine sequence ^a^ (Plain format) |
| **Protein-sol** | - | Multi-epitope vaccine sequence ^a^ (FASTA format) |
| **CamSol-Intrinsic v2.2** | pH: 10 ^b^ | Multi-epitope vaccine sequence ^a^ (FASTA format) |
| **SecretomeP-1.0** | - | Multi-epitope vaccine sequence (FASTA format) |
| **SecretomeP-2.0** | Gram-negative bacteria | Multi-epitope vaccine sequence (FASTA format) |
| **VirulentPred2.0** | - | Multi-epitope vaccine sequence (FASTA format) |
| **VaxiJen v2.0** | Target organism: Bacteria  Threshold: 0.4 | Protein sequence, epitope sequence, multi-epitope vaccine sequence (Plain format) |
| **ANTIGENpro** | - | Multi-epitope vaccine sequence (Plain format) |
| **BLASTp** | Database: Non-redundant protein sequences (nr)  Organism: *H. sapiens* (taxid: 9606)  Algorithm: blastp (protein-protein BLAST)  Max target sequences: 100  Short queries: Activated  Expect threshold: 0.05  Word size: 5  Max matches in a query range: 0  Matrix: BLOSUM62  Gap costs: Existence: 11 Extension: 1  Compositional adjustments: Conditional compositional score matrix adjustment  Filter: Deactivated  Mask: Deactivated | Multi-epitope vaccine sequence (FASTA format) |
| **AllerTOP v2.1** | - | Multi-epitope vaccine sequence (Plain format) |
| **AllerCatPro v2.0** | - | Multi-epitope vaccine sequence (FASTA format) |
| **ToxDL** | - | Multi-epitope vaccine sequence (FASTA format) |
| **ToxinPred2** | Machine learning technique: Hybrid model (RF+BLAST+MERCI)  Threshold value: 0.6 | Multi-epitope vaccine sequence (FASTA format) |
| **PSIPRED V4.02** | Analysis: PSIPRED 4.0 (Predict Secondary Structure) | Multi-epitope vaccine sequence (FASTA format) |
| **JPred4** | Skip searching PDB before prediction: Activated | Multi-epitope vaccine sequence (FASTA format) |
| **Robetta** | Optional parameters: Deactivated | Multi-epitope vaccine sequence (Plain format) |
| **AlphaFold 3** | Entity type: Protein  Copies: 1  Use PDB templates with default cut-off date (30/09/2021): Activated | Multi-epitope vaccine sequence (FASTA format) |
| **GalaxyRefine** | - | Robetta predicted 3D models (PDB file) |
| **PROCHECK** | - | Refined 3D structure of the multi-epitope vaccine (PDB file) |
| **VERIFY3D** | - | Refined 3D structure of the multi-epitope vaccine (PDB file) |
| **ERRAT2** | - | Refined 3D structure of the multi-epitope vaccine (PDB file) |
| **ProSA** | - | Refined 3D structure of the multi-epitope vaccine (PDB file) |
| **Mol* 3D Viewer v5.0.0** | Components: Polymer (Cartoon) / Residue property (Accessible protein residues / Buried protein residues)  Set coloring: Residue property (Secondary structure)  Representation: Ball & Stick / Gaussian surface | Refined 3D structure of the multi-epitope vaccine (PDB file) |
| **CamSol-Structurally corrected v2.3** | pH: 10 ^b^  A (surface patch of size): 10 | Cleaned 3D structure of the multi-epitope vaccine (PDB file) ^c^ |
| **SOLart v1.0** | Chain A: Activated  Organism: *E. coli* / *H. sapiens* | Refined 3D structure of the multi-epitope vaccine (PDB file) |
| **C-ImmSim** | Random seed: 12000  Simulation volume: 10  Simulation steps: 540  Host HLA selection: A0101 (A MHC class I) / B0702 (B MHC class I) / DRB1_0101 (DR MHC class II)  Time step of injection: 3 (injection 1) / 42 (injection 2) / 84 (injection 3)  What to inject: Vaccine (no LPS)  Adjuvant: 100  Num Ag to inject: 1000 | Multi-epitope vaccine sequence (FASTA format) |
| **Population Coverage –**  **IEDB v3.0.2 server** | Number of epitopes: 8  Area: World / East Asia / Northeast Asia / South Asia / Southeast Asia / Southwest Asia / Europe / East Africa / West Africa / Central Africa / North Africa / South Africa / West Indies / North America / Central America / South America / Oceania  Calculation option: Class I separate / Class II separate / Class I and II combined  MHC-restricted alleles for the BEPI-vaccine: HLA-A*01:01,HLA-A*02:01,HLA-A*02:03,HLA-A*02:06,HLA-A*03:01,HLA-A*11:01,HLA-A*26:01,HLA-A*30:01,HLA-A*30:02,HLA-A*31:01,HLA-A*68:01,HLA-A*68:02,HLA-B*07:02,HLA-B*15:01,HLA-B*35:01,HLA-DRB1*01:01,HLA-DRB1*03:01,HLA-DRB1*04:01,HLA-DRB1*04:05,HLA-DRB1*07:01,HLA-DRB1*08:02,HLA-DRB1*09:01,HLA-DRB1*11:01,HLA-DRB1*12:01,HLA-DRB1*13:02,HLA-DRB1*15:01,HLA-DRB3*01:01,HLA-DRB3*02:02,HLA-DRB4*01:01,HLA-DRB5*01:01,HLA-DQA1*05:01,HLA-DQB1*03:01,HLA-DQA1*04:01,HLA-DQB1*04:02,HLA-DQA1*01:02,HLA-DQB1*06:02,HLA-DPA1*02:01,HLA-DPB1*01:01,HLA-DPA1*01:03,HLA-DPB1*02:01,HLA-DPB1*04:01,HLA-DPA1*03:01,HLA-DPB1*04:02,HLA-DPA1*02:01,HLA-DPB1*05:01,HLA-DPB1*14:01  MHC-restricted alleles for the VAXI-vaccine: HLA-A*02:01,HLA-A*02:03,HLA-A*02:06,HLA-A*03:01,HLA-A*11:01,HLA-A*30:01,HLA-A*30:02,HLA-A*31:01,HLA-A*33:01,HLA-A*68:01,HLA-A*68:02,HLA-B*07:02,HLA-B*08:01,HLA-B*57:01,HLA-B*58:01,HLA-DRB1*01:01,HLA-DRB1*04:01,HLA-DRB1*04:05,HLA-DRB1*07:01,HLA-DRB1*08:02,HLA-DRB1*09:01,HLA-DRB1*11:01,HLA-DRB1*13:02,HLA-DRB1*15:01,HLA-DRB3*02:02,HLA-DRB4*01:01,HLA-DRB5*01:01,HLA-DQA1*05:01,HLA-DQB1*03:01,HLA-DQA1*04:01,HLA-DQB1*04:02,HLA-DQA1*01:02,HLA-DQB1*06:02,HLA-DPA1*02:01,HLA-DPB1*01:01,HLA-DPA1*01:03,HLA-DPB1*02:01,HLA-DPB1*04:01,HLA-DPA1*03:01,HLA-DPB1*04:02,HLA-DPA1*02:01,HLA-DPB1*05:01,HLA-DPB1*14:01 | Epitope sequence (Plain format) |
| **MHC-I Binding Predictions v3.1.6** | Prediction method: NetMHCpan-4.1 BA  MHC source species: Human  HLA allele reference set: Activated | Multi-epitope vaccine sequence (FASTA format) |
| **MHC-II Binding Predictions v3.1.12** | Prediction method: NetMHCIIpan-4.0 BA  Species/locus: Human, HLA-DP / HLA-DQ / HLA-DR  Full HLA reference set: Activated  Length: Default 15 | Multi-epitope sequence (FASTA format) |

^a^ 6His-tagged multi-epitope vaccine sequence; ^b^ predicted pI; and ^c^ a preliminary step recommended by the server using the refined PDB file.

**Supplementary Textfiles**

**Supplementary Textfile S1. The amino acid sequences of the BEPI and VAXI multi-epitope vaccines.** Methionine amino acid (blue), B cell epitopes (black), KK linker (yellow), GPGPG linker (orange), PADRE peptide (purple), and 6-his tag (red). The B cell epitopes consecutively belong to the virulence factors MrkA, OmpA, OmpW, LamB, PhoE, FepA and Fiu.

>BEPI multi-epitope vaccine sequence

MGNLLAGATSKQQGYLANTEASGAKKDTGFYGNGFQNNNGPTRNKKTEGSDNVLGSLGSFNVSKKSGGSYTFSSDDTKKYAAKKLKSDNKLGINDKKSRMDEGLAGGTEGIFSSSKKCGGSGRGAIACPPGQSTGSPVGPGPGAKFVAAWTLKAAA(hhhhhh)

>VAXI multi-epitope vaccine sequence

MCQAADGTKQDDVSKKNTCDNVKARAALIKKKFKAGGVDQKVSTKKPQDDYRLEDGAKKSDYDSKDGDKKKRFNYKGEAVSGSKKCGGSGRGAIACPPGQSTGSPVGPGPGAKFVAAWTLKAAA(hhhhhh)

**Supplementary Textfile S2. Optimized DNA sequences of the BEPI and VAXI multi-epitope vaccines for expression in *Escherichia coli* and *Homo sapiens* using JCat and Emboss backtranseq tools**. Start codon (blue), B cell epitopes (black), KK linker (yellow), GPGPG linker (orange), PADRE peptide (purple), 6-his tag (red), and stop codon (blue). The B cell epitopes consecutively belong to the virulence factors MrkA, OmpA, OmpW, LamB, PhoE, FepA and Fiu.

>BEPI multi-epitope vaccine adapted to *E. coli* expression

ATGGGTAACCTGCTGGCTGGTGCTACCTCTAAACAGCAGGGTTACCTGGCTAACACCGAAGCTTCTGGTGCTAAAAAAGACACCGGTTTCTACGGTAACGGTTTCCAGAACAACAACGGTCCGACCCGTAACAAAAAAACCGAAGGTTCTGACAACGTTCTGGGTTCTCTGGGTTCTTTCAACGTTTCTAAAAAATCTGGTGGTTCTTACACCTTCTCTTCTGACGACACCAAAAAATACGCTGCTAAAAAACTGAAATCTGACAACAAACTGGGTATCAACGACAAAAAATCTCGTATGGACGAAGGTCTGGCTGGTGGTACCGAAGGTATCTTCTCTTCTTCTAAAAAATGCGGTGGTTCTGGTCGTGGTGCTATCGCTTGCCCGCCGGGTCAGTCTACCGGTTCTCCGGTTGGTCCGGGTCCGGGTGCTAAATTCGTTGCTGCTTGGACCCTGAAAGCTGCTGCTCACCACCACCACCACCACtaa

>VAXI multi-epitope vaccine adapted to *E. coli* expression

ATGTGCCAGGCTGCTGACGGTACCAAACAGGACGACGTTTCTAAAAAAAACACCTGCGACAACGTTAAAGCTCGTGCTGCTCTGATCAAAAAAAAATTCAAAGCTGGCGGTGTTGACCAGAAAGTAAGCACCAAAAAACCGCAGGACGACTACCGTCTGGAAGACGGTGCTAAAAAATCTGACTACGACTCTAAAGACGGTGACAAAAAAAAACGTTTCAACTACAAAGGCGAGGCTGTAAGCGGTTCTAAAAAATGCGGTGGTTCTGGTCGTGGTGCTATCGCTTGCCCGCCGGGTCAGTCTACCGGTTCTCCGGTTGGTCCGGGTCCGGGTGCTAAATTCGTTGCTGCTTGGACCCTGAAAGCTGCTGCTCACCACCACCACCACCACtaa

>BEPI multi-epitope vaccine adapted to *H. sapiens* expression

ATGGGCAACCTGCTGGCCGGCGCCACCAGCAAGCAGCAGGGCTACCTGGCCAACACCGAGGCCAGCGGCGCCAAGAAGGACACCGGCTTCTACGGCAACGGCTTCCAGAACAACAACGGCCCCACCCGCAACAAGAAGACCGAGGGCAGCGACAACGTGCTGGGCAGCCTGGGCAGCTTCAACGTGAGCAAGAAGAGCGGCGGCAGCTACACCTTCAGCAGCGACGACACCAAGAAGTACGCCGCCAAGAAGCTGAAGAGCGACAACAAGCTGGGCATCAACGACAAGAAGAGCCGCATGGACGAGGGCCTGGCCGGCGGCACCGAGGGCATCTTCAGCAGCAGCAAGAAGTGCGGCGGCAGCGGCCGCGGCGCCATCGCCTGCCCCCCCGGCCAGAGCACCGGCAGCCCCGTGGGCCCCGGCCCCGGCGCCAAGTTCGTGGCCGCCTGGACCCTGAAGGCCGCCGCCtga

>VAXI multi-epitope vaccine adapted to *H. sapiens* expression

ATGTGCCAGGCCGCCGACGGCACCAAGCAGGACGACGTGAGCAAGAAGAACACCTGCGACAACGTGAAGGCCCGCGCCGCCCTGATCAAGAAGAAGTTCAAGGCCGGCGGCGTGGACCAGAAGGTGAGCACCAAGAAGCCCCAGGACGACTACCGCCTGGAGGACGGCGCCAAGAAGAGCGACTACGACAGCAAGGACGGCGACAAGAAGAAGCGCTTCAACTACAAGGGCGAGGCCGTGAGCGGCAGCAAGAAGTGCGGCGGCAGCGGCCGCGGCGCCATCGCCTGCCCCCCCGGCCAGAGCACCGGCAGCCCCGTGGGCCCCGGCCCCGGCGCCAAGTTCGTGGCCGCCTGGACCCTGAAGGCCGCCGCCtga

**Supplementary Figures**

**Supplementary Figure S1. Amino acid types of the final multi-epitope vaccine constructs using PSIPRED V4.02 tool.** (A) BEPI-vaccine construct; and (B) VAXI-vaccine construct.

**Supplementary Figure S2. Additional characteristics of the vaccine proteins predicted by Protein-sol tool.** (A) BEPI-vaccine construct; and (B) VAXI-vaccine construct. From top to bottom, the graphs display deviations from population average of different protein characteristics contributing to solubility, protein net charge subdomains, and protein folding subdomains.

**Supplementary Figure S3. Secondary structure and solvent accessibility prediction of the final multi-epitope vaccine constructs using JPred4 tool.** (A) BEPI-vaccine construct; and (B) VAXI-vaccine construct. The golden square indicates the relative solvent accessibility threshold chosen. Alpha-helix (red tubes), beta-sheet (green arrows), coil (grey line), buried residue (B), and exposed residue (-).

**Supplementary Figure S4. Tertiary structures of the multi-epitope vaccines using the AlphaFold 3 predictor.** (A-B) Visualization of the 3D models of BEPI- and VAXI-vaccine constructs showing color-coded prediction confidence scores.

**Supplementary Figure S5. Validation of the multi-epitope vaccine three-dimensional structures using ERRAT2 and VERIFY3D tools (SAVES v6.1 server).** (A-B) ERRAT2 results; and (C-D) VERIFY3D results for BEPI- and VAXI-vaccine constructs.

**Supplementary Figure S6. Accessible surface area of the final multi-epitope vaccine 3D models using Mol* 3D Viewer v5.0.0.** (A-B) Visualization of the 3D models of BEPI- and VAXI-vaccine constructs after refinement, using the color code of secondary-structure regions and representing accessible and buried protein residues.

**Supplementary Figure S7. Solubility profiles of the BEPI and VAXI multi-epitope vaccines using CamSol v2.2/v2.3 and SOLart v1.0 tools**. (A-B) BEPI-vaccine construct; and (C-D) VAXI-vaccine construct. Solubility predictions were performed considering the final 3D structure models (without 6-his tag). The golden square indicates the top six structure-based features for the SOLart solubility algorithm according to solvent accessibility potential (ssa, saa and sa), backbone torsion angle potential (sst), and inter-residue distance potential (sd and sds).

**Supplementary Figure S8. Simulated immune response upon BEPI and VAXI multi-epitope vaccines administration using C-ImmSim server.** (A-B) Cytotoxic T cell populations; (C-D) Cytotoxic T cell populations per state; (E-F) Dendritic cell populations per state; and (G-H) Macrophage populations per state for BEPI- and VAXI-vaccine constructs.
